# Prediabetes Phenotypes and Adiposity Patterns: Findings From a Population‐Based Study

**DOI:** 10.1155/jdr/1147673

**Published:** 2026-07-20

**Authors:** Jincheng Rong, Mandy Ho, Sarah Garnett, Pui Hing Chau

**Affiliations:** ^1^ School of Nursing, LKS Faculty of Medicine, The University of Hong Kong, Hong Kong SAR, China, hku.hk; ^2^ Children’s Hospital Westmead Clinical School, The University of Sydney, Westmead, New South Wales, Australia, sydney.edu.au; ^3^ Kids Research, Sydney Children’s Hospital Network, Westmead, New South Wales, Australia, schn.health.nsw.gov.au

**Keywords:** diabetes prevention, fat distribution, impaired fasting glucose, impaired glucose tolerance, obesity, prediabetes

## Abstract

**Background:**

Prediabetes is a heterogeneous condition encompassing three glucose‐defined phenotypes (isolated impaired fasting glucose [i‐IFG], isolated impaired glucose tolerance [i‐IGT], and IFG + IGT), with distinct pathophysiological mechanisms. This study assessed the associations of weight status (body mass index [BMI]), general adiposity (fat mass index [FMI]), total lean mass (lean mass index [LMI]), and body fat distribution (waist circumference [WC] and DEXA‐derived appendicular, gynoid, abdominal and visceral adiposity) with prediabetes phenotypes.

**Methods:**

This cross‐sectional study included 3225 adults without diabetes who had complete data on fasting and 2‐h plasma glucose, anthropometric measures (BMI and WC), and DEXA‐derived measures (FMI, LMI, and percentages of total fat in appendicular, gynoid, abdominal and visceral regions) from the National Health and Nutrition Examination Survey 2011–2016. BMI and WC were classified according to WHO criteria. DEXA‐derived measures were classified using sex‐specific tertiles. Glycemic status was classified as normoglycemia, i‐IFG, i‐IGT, or IFG + IGT based on ADA criteria. Logistic regression and restricted cubic spline analyses were performed.

**Results:**

The weighted mean (SD) age was 37.16 (12.15) years, and 49.5% of participants were male. Overall, 59.6%, 29.1%, 4.2%, and 7.0% had normoglycemia, i‐IFG, i‐IGT, and IFG + IGT, respectively. Compared with individuals with normal BMI, those with overweight or obesity had higher odds of i‐IFG (overweight: OR = 1.56 [1.19, 2.03]; obesity: OR = 2.73 [2.09, 3.56]) and IFG + IGT (overweight: OR = 2.81 [1.61, 4.91]; obesity: OR = 6.72 [4.03, 11.22]), whereas both underweight (OR = 3.22 [1.21, 8.58]) and obesity (OR = 2.57 [1.64, 4.04]) were associated with higher odds of i‐IGT, indicating a U‐shaped relationship between BMI and i‐IGT (*p*_non−linearity_ = 0.006). Higher FMI and LMI were associated with higher odds of all three phenotypes (all p_T3vs.T1_ < 0.05). Compared with normal WC, very‐high‐risk central obesity was associated with higher odds of all three phenotypes (all *p* < 0.05). Higher proportions of abdominal or visceral fat and lower proportions of appendicular or gynoid fat were associated with higher odds of i‐IFG and IFG + IGT (all p_T3vs.T1_ < 0.001). For i‐IGT, only gynoid fat showed an inverse association (p_T3vs.T1_ = 0.002).

**Conclusion:**

Adiposity patterns differed across prediabetes phenotypes. These findings provide insights for tailoring intervention strategies by prediabetes phenotype to optimize diabetes prevention.

## 1. Introduction

Type 2 diabetes is a leading global health concern with substantial impacts on morbidity and mortality, making its prevention a critical public health priority [1]. Prediabetes, a precursor to Type 2 diabetes, provides a window of opportunity for interventions to prevent or delay diabetes onset. Current clinical guidelines recommend lifestyle modification, with an emphasis on weight management, as the first‐line intervention for individuals with prediabetes [2]. However, prediabetes is a heterogeneous condition encompassing three phenotypes: isolated impaired fasting glucose (i‐IFG), isolated impaired glucose tolerance (i‐IGT), and combined IFG and IGT (IFG + IGT) [3]. Existing literature has demonstrated phenotypic differences in prevalence, pathophysiological and etiological mechanisms, and risk of progression to diabetes. For example, hepatic insulin resistance is the primary defect underlying IFG, whereas IGT mainly involves muscle insulin resistance [3–5]. Recent meta‐analyses have also reported differential responses to lifestyle interventions across distinct prediabetes phenotypes [6, 7]. This highlights the need to better understand phenotype‐specific risk factors to inform tailored lifestyle modification strategies and improve diabetes prevention efficacy.

Obesity is a well‐established risk factor for insulin resistance and Type 2 diabetes [8]. Body mass index (BMI) is the most widely used obesity indicator, but it has a well‐recognized limitation—the inability to differentiate between fat and muscle mass. Mechanistic research has suggested that skeletal muscle may differentially influence fasting plasma glucose (FPG) and 2‐h post‐load plasma glucose (2 h‐PG) regulation, serving as the primary site for postprandial glucose disposal while playing a relatively minor role during fasting [3]. This implies that the associations with weight status and body composition may differ across distinct prediabetes phenotypes. However, evidence remains limited on the associations of weight status, total adiposity, and lean mass with distinct prediabetes phenotypes.

Body fat distribution also plays an important role in the development of Type 2 diabetes [9]. Appendicular fat, which surrounds or infiltrates skeletal muscle, may contribute to muscle insulin resistance [10]. Abdominal fat, particularly visceral adipose tissue (VAT) located inside the abdominal cavity, is particularly detrimental to hepatic glucose and lipid metabolism due to its drainage via the portal vein [11–13]. Despite these mechanistic insights, only a few studies have investigated the associations between body fat distribution and distinct prediabetes phenotypes. Two studies have examined the associations with VAT, measured using imaging techniques, but reported mixed findings [14, 15]. To our knowledge, no studies have examined the associations of appendicular, gynoid (hip and upper thigh), or total abdominal adipose tissue with distinct prediabetes phenotypes.

Using anthropometric and dual‐energy X‐ray absorptiometry (DEXA)‐derived whole body and regional body composition data from a nationally representative US sample, this study aimed to comprehensively assess the associations of weight status (BMI), general adiposity (fat mass index [FMI]), total lean mass (lean mass index [LMI]), and body fat distribution (waist circumference [WC] and DEXA‐derived percentages of total fat in appendicular, gynoid, abdominal, and visceral regions) with prediabetes phenotypes (i‐IFG, i‐IGT, and IFG + IGT). This knowledge may provide valuable insights into tailored intervention strategies for distinct prediabetes phenotypes, complementing current weight management recommendations for diabetes prevention [2].

## 2. Methods

### 2.1. Study Design

This cross‐sectional study used data from the US National Health and Nutrition Examination Survey (NHANES), a series of surveys conducted every 2 years to evaluate the health and nutritional status of children and adults in the US population [16]. Three waves (2011–2012, 2013–2014, and 2015–2016), comprising 29,902 participants, were selected because they provided both oral glucose tolerance test (OGTT) and DEXA data. This study followed the Strengthening the Reporting of Observational Studies in Epidemiology guideline [17].

### 2.2. Participants

Individuals were excluded if they (1) were aged < 18 years; (2) had missing data on anthropometric measures (weight, height, or WC), DEXA‐derived measures (total fat mass, total lean mass, arm fat mass, leg fat mass, abdominal fat mass, gynoid fat mass, or visceral fat mass), or glycemic measurements (FPG or 2 h‐PG); or (3) had diabetes, defined by self‐report or blood tests (FPG ≥ 7.0 mmol/L or 2 h‐PG ≥ 11.1 mmol/L), based on the American Diabetes Association criteria [18].

### 2.3. Anthropometric Measures and DEXA‐Derived Body Composition

Body weight, standing height, and WC were measured by trained health technicians following the standard procedure manual [16]. DEXA was performed in a subset of individuals aged 8–59 years to determine body composition, including whole‐body and regional fat, lean, and bone mass, using standard techniques [16]. Detailed information on weight status, general adiposity, total lean mass, and body fat distribution indicators is provided in Table S1.

BMI was classified into four weight status categories according to the ethnic‐specific World Health Organization (WHO) criteria [19]: underweight (non‐Asian and Asian: < 18.5 kg/m^2^), normal weight (non‐Asian: 18.5–24.9 kg/m^2^; Asian: 18.5–22.9 kg/m^2^), overweight (non‐Asian: 25.0–29.9 kg/m^2^; Asian: 23.0–24.9 kg/m^2^), and obesity (non‐Asian: ≥ 30.0 kg/m^2^; Asian: ≥ 25.0 kg/m^2^). WC was classified according to the WHO sex‐specific cut‐offs: normal WC (< 80 cm in females and < 94 cm in males), high‐risk central obesity (80 to < 88 cm in females and 94 to < 102 cm in males), and very‐high‐risk central obesity (≥ 88 cm in females and ≥ 102 cm in males) [20].

General adiposity was measured using FMI, calculated as total fat mass divided by height squared [21]. Total lean mass was assessed using LMI, calculated as total lean mass divided by height squared [21]. Body fat distribution was expressed as regional fat mass relative to total fat mass, including the appendicular, gynoid, abdominal, and visceral regions. Peripheral fat refers to appendicular and gynoid adipose tissues, whereas central fat refers to abdominal and visceral adipose tissues. Preferential fat deposition refers to the tendency to accumulate body fat in a specific region, as indicated by a higher proportion of total fat stored in that area.

### 2.4. Plasma Glucose/Insulin and Glycemic Status Classification

The 75‐g OGTT was performed following the laboratory procedure manual [16]. Fasting blood samples were first collected to measure FPG and fasting insulin. Participants were then asked to drink 75 g of the Trutol solution, and a second blood sample was collected to measure 2 h‐PG.

After excluding those with diabetes, the remaining individuals were classified into: (1) normoglycemia: FPG < 5.6 mmol/L and 2 h‐PG < 7.8 mmol/L; (2) i‐IFG: FPG 5.6–6.9 mmol/L and 2 h‐PG < 7.8 mmol/L; (3) i‐IGT: FPG < 5.6 mmol/L and 2 h‐PG 7.8–11.0 mmol/L; and (4) IFG + IGT: FPG 5.6–6.9 mmol/L and 2 h‐PG 7.8–11.0 mmol/L [18]. The Homeostatic Model Assessment for Insulin Resistance (HOMA‐IR) was calculated as FPG (mmol/L) × FI (*μ*U/mL)/22.5 [22].

### 2.5. Covariates

Covariates were selected a priori as potential confounders based on their associations with both adiposity and glycemic status [23–25]. Demographic variables included age, sex, and ethnicity. As DEXA was only performed in a subset of individuals aged 8–59 years, age was classified into two groups: 18–44 years and 45–59 years. Ethnicity was grouped as Hispanic, including Mexican American and other Hispanics; non‐Hispanic White; non‐Hispanic Black; non‐Hispanic Asian; and others. Education and poverty income ratio (PIR) were used as indicators of socioeconomic status, which may influence adiposity and glycemic status through health literacy, healthcare access, food choices, opportunities for physical activity (PA), and broader social and environmental conditions [25]. Education was grouped as lower than high school, high school or equivalent, and college or higher. PIR was categorized as < 1.0, 1.0–3.0, and > 3.0. PA data were collected by the Global Physical Activity Questionnaire and expressed as metabolic equivalent of task (MET) minutes/week. Individuals with ≥ 600 MET‐min/week were classified as physically active according to the WHO recommendations [26]. A healthy level of alcohol intake was defined as ≤ 1 drink/day for females and ≤ 2 drinks/day for males, and one drink contains 14 g of ethanol according to the US guidelines [27]. Smoking status was classified as nonsmoker (< 100 cigarettes in life) or smoker [28]. Data on family history of diabetes were self‐reported.

### 2.6. Statistical Analysis

Considering the complex multistage sampling design of NHANES, all analyses incorporated sample weights, strata, and primary sampling units to facilitate generalization according to the analytic guidelines [16]. The sample weight was calculated as the OGTT sample weight divided by the number of waves used [16].

Normality was assessed using the Kolmogorov–Smirnov test. Continuous data were presented as weighted mean with standard deviation (SD) if normally distributed and as weighted median with interquartile range (IQR) if non‐normally distributed. Analysis of variance (ANOVA) and the Kruskal–Wallis test were performed to compare normally and non‐normally distributed continuous variables, respectively. Categorical data were presented as unweighted frequency with weighted percentages and compared using the chi‐square test.

Weighted logistic regression models were used to estimate odds ratios (ORs) with 95% confidence intervals (CIs) for each prediabetes phenotype, compared with normoglycemia, in relation to weight status, general adiposity, total lean mass, and body fat distribution. These exposure variables were analyzed categorically. BMI was categorized based on the WHO ethnic‐specific cutoffs, with normal weight as the reference [19]. WC was categorized based on the WHO sex‐specific cutoffs, with normal WC as the reference [20]. Given sex differences in body composition and body fat distribution [29], DEXA‐derived variables (FMI, LMI, appendicular adipose tissue [%], abdominal adipose tissue [%], gynoid adipose tissue [%], and VAT [%]) were categorized using sex‐specific tertiles, with the first tertile as the reference. Models were adjusted for age, sex, ethnicity, education, PIR, PA, smoking, alcohol consumption, and family history of diabetes. Models for body fat distribution variables were additionally adjusted for height. Multicollinearity was assessed using the variance inflation factor (VIF), with a VIF > 10 indicating significant multicollinearity. Restricted cubic spline (RCS) analyses were used to assess non‐linear associations. According to the WHO criteria, the same BMI cutoffs were used for males and females, with knots set at 18.5, 22.9, 24.9, and 30.0 kg/m^2^ [19]. Given sex differences in body fat accumulation and distribution [29], sex‐specific analyses were performed with knots at the 10th, 50th, and 90th percentiles. Non‐linearity was assessed using the Wald test.

Exploratory analyses were performed to provide mechanistic insight. First, the main models were additionally adjusted for HOMA‐IR to examine whether insulin resistance may partly account for the associations between adiposity indicators and prediabetes phenotypes. Second, given the correlation between total lean mass and fat mass, the LMI models were further adjusted for FMI to assess whether the associations between LMI and prediabetes phenotypes were independent of total adiposity.

Stratified analyses were performed by sex (female vs. male), age (18–44 vs. 45–59 years), ethnicity (Hispanic vs. non‐Hispanic White vs. others), and weight status (non‐overweight vs. overweight or obesity). Interactions were assessed by the likelihood ratio test. Missing data in covariates were imputed using multiple imputation by chained equations. All statistical analyses were performed in R Version 4.4.0. Statistical significance was set at a two‐sided *p* < 0.05.

## 3. Results

### 3.1. Participant Characteristics

A flowchart of the participant selection process is presented in Figure S1. A total of 3225 adults were included in this study. The weighted mean (SD) age was 37.16 (12.15) years, and 49.5% were male. The weighted percentages of individuals with normoglycemia, i‐IFG, i‐IGT, and IFG + IGT were 59.6%, 29.1%, 4.2%, and 7.0%, respectively. Demographic, lifestyle, and clinical characteristics of the study sample are summarized by glycemic status in Table 1. Given sex differences in body fat accumulation and distribution, sex‐specific anthropometric and DEXA‐derived characteristics are presented by glycemic status in Table 2. Generally, both males and females with prediabetes tended to have higher BMI, FMI, LMI, and WC; higher proportions of total fat in abdominal and visceral regions; and lower proportions of total fat in gynoid and appendicular regions compared with their counterparts with normoglycemia (all *p* < 0.001).

**Table 1:** Characteristics of the study population by glycemic status in the NHANES 2011–2016.

Variables^a^	Normal	i‐IFG	i‐IGT	IFG + IGT	*p* value^b^
Unweighted no. of individuals (%)	1,897 (58.8)	946 (29.3)	150 (4.7)	232 (7.2)	NA
Weighted no. of individuals (%)	76,769,216 (59.6)	37,506,911 (29.1)	5,378,290 (4.2)	9,053,377 (7.0)	NA
Demographic					
Age group, *N* (%)					**< 0.001**
18–44 years	1,479 (75.5)	592 (61.0)	87 (60.3)	118 (46.1)	
45–59 years	418 (24.5)	354 (39.0)	63 (39.7)	114 (53.9)	
Sex					**< 0.001**
Female	1,064 (57.2)	332 (37.2)	87 (56.4)	106 (45.4)	
Male	833 (42.8)	614 (62.8)	63 (43.6)	126 (54.6)	
Ethnicity, *N* (%)					**0.004**
Hispanic	444 (16.0)	293 (19.7)	40 (18.3)	81 (22.9)	
Non‐Hispanic White	714 (62.1)	346 (62.7)	47 (56.8)	69 (57.6)	
Non‐Hispanic Black	409 (13.4)	139 (8.9)	25 (11.3)	35 (9.6)	
Non‐Hispanic Asian	269 (5.9)	131 (5.4)	33 (10.7)	43 (8.5)	
Other races	61 (2.7)	37 (3.3)	5 (2.8)	4 (1.4)	
Education, *N* (%)					0.452
Lower than high school	337 (14.5)	210 (15.7)	36 (19.2)	62 (18.6)	
High school or equivalent	400 (19.8)	232 (23.1)	30 (19.7)	52 (24.0)	
College or higher	1,160 (65.7)	504 (61.2)	84 (61.2)	118 (57.4)	
Poverty impact ratio, *N* (%)					0.314
< 1.0	437 (18.7)	225 (17.1)	33 (17.1)	61 (20.7)	
1.0 to < 3.0	664 (35.2)	340 (36.8)	51 (36.1)	69 (26.7)	
≥ 3.0	665 (46.0)	312 (46.1)	54 (46.8)	85 (52.6)	
Lifestyle					
Smoking status, *N* (%)^c^					**0.015**
Non‐smoker	1,192 (63.3)	527 (56.8)	100 (68.0)	137 (50.7)	
Smoker	632 (36.7)	406 (43.2)	48 (32.0)	93 (49.3)	
Drinking status, *N* (%)^d^					**0.082**
Healthy	1,702 (92.9)	817 (89.6)	129 (86.4)	207 (87.7)	
Unhealthy	120 (7.1)	79 (10.4)	13 (13.6)	18 (12.3)	
Physical activity status, *N* (%)^e^					**0.001**
Inactive	507 (26.4)	282 (28.1)	57 (41.1)	98 (43.7)	
Active	1,383 (73.6)	658 (71.9)	92 (58.9)	134 (56.3)	
Clinical and biochemistry					
FPG, mg/dL, median (IQR)	92.00 (88.00, 96.00)	105.00 (102.00, 108.00)	94.00 (89.00, 98.00)	108.00 (103.00, 114.00)	**< 0.001**
2 h‐PG, mg/dL, median (IQR)	92.00 (78.00, 107.00)	103.00 (88.00, 118.00)	155.00 (146.00, 170.00)	158.00 (147.00, 176.00)	**< 0.001**
HOMA‐IR	1.66 (1.07, 2.61)	2.78 (1.89, 4.29)	2.33 (1.18, 3.54)	4.10 (2.42, 6.33)	**< 0.001**
Family history of diabetes, *N* (%)	563 (32.0)	324 (34.4)	55 (38.9)	100 (50.8)	**< 0.001**

*Note:* Bold values indicate statistical significance at *p* < 0.05.

Abbreviations: FPG = fasting plasma glucose; HbA1c = glycated hemoglobin A1c; IFG + IGT = impaired both fasting plasma glucose and glucose tolerance; I‐IFG = isolated‐impaired plasma glucose; I‐IGT = isolated‐impaired glucose tolerance; IQR = interquartile range; 2 h‐PG = 2‐hour post‐load plasma glucose.

^a^Continuous data was presented as weighted median with IQR. Categorical data was presented as unweighted frequency with weighted percentages.

^b^The Kruskal–Wallis test and the chi‐square test were performed to compare continuous and categorical variables across glycemic status, respectively.

^c^Smoking status was classified as non‐smoker (< 100 cigarettes in life) or smoker.

^d^A healthy level of alcohol intake was defined as ≤ 1 drink/day for females and ≤ 2 drinks/day for males, and one drink contains 14 g of ethanol according to the US guidelines.

^e^Individuals with ≥ 600 MET‐min/week were classified as physically active according to the WHO recommendations.

**Table 2:** Anthropometric and dual‐energy X‐ray absorptiometry characteristics of the study population by sex and glycemic status.

Anthropometric and DEXA characteristics	Females					Male				
	Normal	i‐IFG	i‐IGT	IFG + IGT	*p* value^b^	Normal	i‐IFG	i‐IGT	IFG + IGT	*p* value^b^
BMI, kg/m^2^, median (IQR)	25.40 (22.10, 30.70)	29.00 (25.10, 35.50)	28.40 (22.80, 32.30)	30.60 (27.60, 34.70)	< 0.001	26.40 (23.00, 29.40)	28.20 (24.80, 31.80)	28.20 (24.00, 31.20)	29.60 (27.10, 33.90)	< 0.001
FMI, kg/m^2^, median (IQR)	9.68 (7.28, 12.94)	12.09 (9.26, 15.70)	11.57 (7.51, 13.28)	12.89 (11.25, 14.86)	< 0.001	6.68 (5.00, 8.54)	7.83 (6.00, 9.69)	8.11 (5.77, 10.46)	8.80 (7.44, 10.66)	< 0.001
LMI, kg/m^2^, median (IQR)	15.38 (14.00, 17.23)	16.57 (14.83, 19.18)	16.18 (13.97, 18.57)	17.76 (15.72, 19.46)	< 0.001	18.79 (17.06, 20.44)	19.49 (17.81, 21.35)	19.82 (17.10, 21.03)	20.85 (18.81, 22.62)	< 0.001
WC, cm, median (IQR)	88.30 (78.80, 99.00)	98.10 (88.40, 109.00)	95.60 (80.10, 106.80)	102.20 (93.20, 114.50)	< 0.001	93.10 (83.50, 102.60)	99.00 (90.40, 107.40)	99.20 (88.50, 107.40)	104.50 (97.20, 114.10)	< 0.001
Appendicular adipose tissue, %, median (IQR)^a^	52.52 (49.08, 56.07)	49.33 (45.69, 53.33)	50.55 (47.30, 54.46)	49.07 (44.99, 51.67)	< 0.001	47.05 (43.70, 50.45)	44.66 (41.52, 48.33)	45.29 (42.21, 49.06)	42.21 (40.25, 45.36)	< 0.001
Gynoid adipose tissue, %, median (IQR)^a^	7.53 (6.69, 8.31)	7.86 (6.95, 8.80)	7.88 (7.12, 8.46)	8.31 (7.59, 8.84)	< 0.001	6.85 (5.65, 7.71)	7.48 (6.64, 8.16)	7.30 (6.18, 7.96)	8.00 (7.38, 8.96)	< 0.001
Abdominal adipose tissue, %, median (IQR)^a^	18.90 (17.22, 20.41)	17.49 (15.98, 19.24)	17.78 (16.42, 18.97)	16.67 (15.56, 18.09)	< 0.001	16.92 (15.77, 18.13)	16.29 (14.97, 17.58)	16.01 (14.92, 17.40)	15.29 (14.32, 16.56)	< 0.001
Visceral adipose tissue, %, median (IQR)^a^	1.21 (0.87, 1.65)	1.55 (1.17, 2.02)	1.51 (1.14, 1.76)	1.88 (1.56, 2.25)	< 0.001	1.93 (1.55, 2.29)	2.08 (1.63, 2.52)	2.13 (1.60, 2.63)	2.50 (1.80, 3.28)	< 0.001

Abbreviations: BMI = body mass index; FMI = fat mass index; IFG + IGT = impaired both fasting plasma glucose and glucose tolerance; I‐IFG = isolated‐impaired plasma glucose; I‐IGT = isolated‐impaired glucose tolerance; IQR = interquartile range; LMI = lean mass index; WC = waist circumference.

^a^DEXA‐derived body fat distribution variables were expressed as regional fat mass relative to total body fat mass, including appendicular adipose tissue (%), gynoid adipose tissue (%), abdominal adipose tissue (%), and visceral adipose tissue (%).

^b^The Kruskal–Wallis test and the chi‐square tests were performed to compare continuous and categorical variables across glycemic status, respectively.

### 3.2. Associations Between Weight Status and Prediabetes Phenotypes

**Figure 1:**
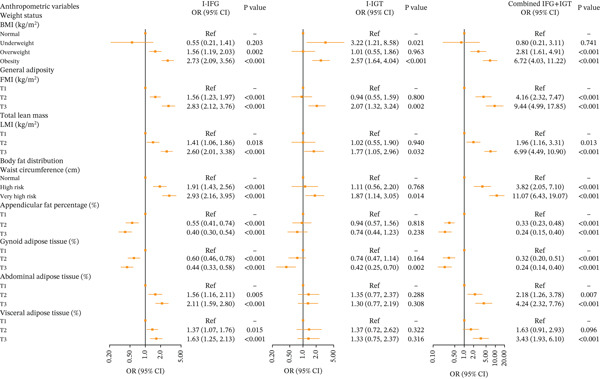
Logistic regression for the associations of weight status, general adiposity, total lean mass, and body fat distribution with three prediabetes phenotypes. All analyses were weighted and accounted for the complex survey design. Models were adjusted for age, sex, ethnicity, education, PIR, physical activity, smoking, alcohol drinking, and family history of diabetes. Models for body fat distribution variables were additionally adjusted for height. DEXA‐derived body fat distribution variables were expressed as regional fat mass relative to total body fat mass, including appendicular adipose tissue (%), abdominal adipose tissue (%), gynoid adipose tissue (%), and visceral adipose tissue (%). BMI was categorized based on the WHO ethnic‐specific cutoffs. WC was categorized based on the WHO sex‐specific cutoffs. Categorization of DEXA‐derived measures was using sex‐specific tertiles. BMI = body mass index; CI = confidence interval; FMI = fat mass index; IFG + IGT = impaired both fasting plasma glucose and glucose tolerance; I‐IFG = isolated‐impaired fasting glucose; I‐IGT = isolated‐impaired glucose tolerance; LMI = lean mass index; OR = odds ratio; Ref = reference; T1 = the first tertile; T2 = the second tertile; T3 = the third tertile; WC = waist circumference.

Compared with individuals with normal BMI, those with overweight or obesity had higher odds of i‐IFG (overweight: OR = 1.56 [1.19, 2.03]; obesity: OR = 2.73 [2.09, 3.56]) and IFG + IGT (overweight: OR = 2.81 [1.61, 4.91]; obesity: OR = 6.72 [4.03, 11.22]), whereas underweight was not significantly associated with i‐IFG (OR = 0.55 [0.21, 1.41]) or IFG + IGT (OR = 0.80 [0.21, 3.11]). For i‐IGT, compared with individuals with normal BMI, those with underweight (OR = 3.22 [1.21, 8.58]) or obesity (OR = 2.57 [1.64, 4.04]) had higher odds, whereas no significant association was observed among those with overweight (OR = 1.01 [0.55, 1.86]; Figure 1).

**Figure 2:**
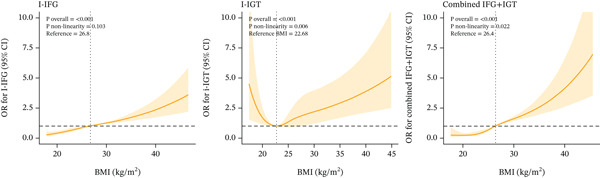
Restricted cubic spline analyses for the associations between body mass index and three prediabetes phenotypes. The solid curved lines represent odds ratios for three prediabetes phenotypes, and the shading areas represent 95% confidence intervals. All analyses were weighted and accounted for the complex survey design. Models were adjusted for age, sex, ethnicity, education, poverty income ratio, physical activity, smoking, alcohol intake, and family history of diabetes.

RCS analyses identified a significant U‐shaped relationship between BMI and odds of i‐IGT with the lowest point at 22.68 kg/m^2^ (*p*_non−linearity_ = 0.006). A positive non‐linear monotonic relationship was identified between BMI and odds of IFG + IGT (*p*_non−linearity_ = 0.022). No evident non‐linear relationship was identified between BMI and odds of i‐IFG (*p*_non−linearity_ = 0.103; Figure 2).

### 3.3. Associations Between General Adiposity and Prediabetes Phenotypes

Similar to BMI, individuals in the third tertile (vs. the first tertile) of FMI had higher odds of i‐IFG (OR_T3 vs.T1_ = 2.83 [2.12, 3.76]), i‐IGT (OR_T3 vs.T1_ = 2.07 [1.32, 3.24]), and IFG + IGT (OR_T3 vs.T1_ = 9.44 [4.99, 17.85]; Figure 1). We identified positive non‐linear monotonic relationships of FMI with odds of i‐IFG in males (*p*_non−linearity_ = 0.006) and with odds of IFG + IGT in both sexes (*p*_non−linearity_ = 0.005 for females; *p*_non−linearity_ = 0.027 for males). No other evident non‐linear relationships were identified between FMI and odds of three prediabetes phenotypes (*p*_non−linearity_ > 0.05; Figure S2).

### 3.4. Associations Between Total Lean Mass and Prediabetes Phenotypes

Similarly, individuals in the third tertile (vs. the first tertile) of LMI had higher odds of i‐IFG (OR_T3 vs.T1_ = 2.60 [2.01, 3.38]), i‐IGT (OR_T3 vs.T1_ = 1.77 [1.05, 2.96]), and IFG + IGT (OR_T3 vs.T1_ = 6.99 [4.49, 10.90]; Figure 1). No evident non‐linear relationships were identified between LMI and odds of three prediabetes phenotypes (all *p*_non−linearity_ > 0.05; Figure S2).

### 3.5. Associations Between Body Fat Distribution and Prediabetes Phenotypes

In the peripheral regions, individuals in the third tertile (vs. the first tertile) of the percentage of DEXA‐derived appendicular adipose tissue relative to total fat mass had lower odds of i‐IFG (OR_T3 vs.T1_ = 0.40 [0.30, 0.54]) and IFG + IGT (OR_T3 vs.T1_ = 0.24 [0.15, 0.40]), but not i‐IGT (OR_T3 vs.T1_ = 0.74 [0.44, 1.23]). Inverse associations were similarly identified between the percentage of DEXA‐derived gynoid adipose tissue relative to total fat mass and odds of three prediabetes phenotypes (i‐IFG: OR_T3 vs.T1_ = 0.44 [0.33, 0.58]; i‐IGT: OR_T3 vs.T1_ = 0.42 [0.25, 0.70]; IFG + IGT : OR_T3 vs.T1_ = 0.24 [0.14, 0.40]; Figure 1).

In contrast to the peripheral regions, the percentage of DEXA‐derived abdominal adipose tissue relative to total fat mass was positively associated with odds of i‐IFG (OR_T3 vs.T1_ = 2.11 [1.59, 2.80]) and IFG + IGT (OR_T3 vs.T1_ = 4.24 [2.32, 7.76]), but not with i‐IGT (OR_T3 vs.T1_ = 1.30 [0.77, 2.19]). Similarly, positive associations were observed between the percentage of DEXA‐derived VAT relative to total fat mass and odds of i‐IFG (OR_T3 vs.T1_ = 1.63 [1.25, 2.13]) and IFG + IGT (OR_T3 vs.T1_ = 3.43 [1.93, 6.10]), whereas no evident association was observed with i‐IGT (OR_T3 vs.T1_ = 1.33 [0.75, 2.37]; Figure 1).

A positive non‐linear monotonic relationship was observed between the percentage of DEXA‐derived VAT relative to total fat mass and odds of IFG + IGT (*p*_non−linearity_ = 0.001). No other evident non‐linear relationships were identified between DEXA‐derived body fat distribution indicators and odds of three prediabetes phenotypes (all *p*_non−linearity_ > 0.05; Figure S2).

Using WC as an anthropometric measure of central obesity, compared with individuals with normal WC, those with high‐risk central obesity had higher odds of i‐IFG (OR = 1.91 [1.43, 2.56]) and IFG + IGT (OR = 3.82 [2.05, 7.10]), whereas those with very‐high‐risk central obesity had higher odds of all three phenotypes (i‐IFG: OR = 2.93 [2.16, 3.95]; i‐IGT: OR = 1.87 [1.14, 3.05]; IFG + IGT: OR = 11.07 [6.43, 19.07]; Figure 1). We identified positive non‐linear monotonic associations of WC with odds of i‐IFG in males (*p*_non−linearity_ = 0.027) and with odds of IFG + IGT in females (*p*_non−linearity_ = 0.040). No other evident non‐linear associations were identified between WC and odds of the three prediabetes phenotypes (all *p*_non−linearity_ > 0.05; Figure S2).

### 3.6. Exploratory Analyses

As expected, additional adjustment for HOMA‐IR attenuated most observed associations between adiposity indicators and prediabetes phenotypes (Table S2). After further adjustment for FMI, the associations of LMI with i‐IFG (OR_T3 vs.T1_ = 1.51 [1.01, 2.24]) and IFG + IGT (OR_T3 vs.T1_ = 4.08 [2.43, 6.85]) were attenuated but remained significant, whereas the association with i‐IGT became nonsignificant (OR_T3 vs.T1_ = 1.34 [0.68, 2.64]; Table S3).

### 3.7. Stratified Analyses

Stratified analyses were performed by sex (Table S4), age group (Table S5), ethnicity (Table S6), and weight status (Table S7). Significant interactions were identified between the percentage of VAT and sex for odds of i‐IFG (OR_T3 vs.T1_ = 2.45 [1.34, 4.46] for females, OR_T3 vs.T1_ = 1.37 [0.98, 1.91] for males; *p*_interaction_ = 0.018) and for odds of i‐IGT (OR_T3 vs.T1_ = 1.66 [0.83, 3.29] for females, OR_T3 vs.T1_ = 1.03 [0.42, 2.52] for males; *p*_interaction_ = 0.030). In addition, a significant interaction was observed between the percentage of gynoid adipose tissue and age group for the odds of IFG + IGT (*p*_interaction_ = 0.021). Among individuals aged 18–44 years, the OR_T3 vs. T1_ for IFG + IGT was 0.35 (0.17, 0.70), whereas the OR_T3 vs. T1_ was 0.08 (0.03, 0.20) among those aged 45–59 years. No other significant interactions were identified (all *p*_interaction_ > 0.05).

## 4. Discussion

To our knowledge, this is the first study to examine the associations of both conventional anthropometric measures and DEXA‐derived measures of general adiposity, lean mass, and body fat distribution with three glucose‐defined prediabetes phenotypes—i‐IFG, i‐IGT, and IFG + IGT—in a large, nationally representative sample. Using ethnicity‐specific BMI cut‐offs, both overweight and obesity were associated with higher odds of i‐IFG and IFG + IGT, whereas both underweight and obesity were associated with higher odds of i‐IGT, suggesting a U‐shaped association between BMI and i‐IGT. Elevated FMI and LMI were associated with higher odds of all three phenotypes. Preferential central fat deposition, characterized by a higher proportion of total body fat in abdominal or visceral regions and a lower proportion in appendicular or gynoid regions, was associated with higher odds of i‐IFG and IFG + IGT. In contrast, only a higher proportion of gynoid fat was inversely associated with i‐IGT. These patterns were broadly consistent across stratified analyses by sex, age, ethnicity, and weight status.

Compared with normal BMI, obesity was consistently associated with higher odds of all three prediabetes phenotypes, supporting obesity prevention and management as a public health priority for reducing the burden of prediabetes and subsequent diabetes. Notably, overweight was associated with i‐IFG and IFG + IGT, but not i‐IGT. A previous study from Bangladesh using the same WHO ethnicity‐specific BMI cut‐offs reported a similar pattern, with overweight associated with an approximately twofold higher prevalence of IFG but not IGT [30]. These findings suggest that overweight may be more strongly related to IFG‐related phenotypes, with or without IGT, than to isolated IGT.

A novel finding was that individuals with underweight had more than a threefold higher odds of i‐IGT than those with normal weight. This association may reflect unmeasured aspects of skeletal muscle health, including muscle mass and quality. Because skeletal muscle is the major site of postprandial glucose disposal, whereas fasting glucose disposal occurs largely in insulin‐independent tissues such as the brain, skeletal muscle health may be more relevant to 2 h‐PG than to FPG regulation. Current prediabetes screening programs mainly target individuals with overweight or obesity [31]. Our findings therefore raise concerns that underweight individuals at risk of i‐IGT may be missed. Further studies are needed to confirm this association and to clarify whether strategies targeting skeletal muscle are effective in this population.

Beyond BMI, general adiposity measured by DEXA‐derived FMI was associated with higher odds of all three prediabetes phenotypes, with stronger associations for i‐IFG and IFG + IGT. This finding partly aligns with a previous study reporting positive associations between FMI and the odds of i‐IFG and IFG + IGT, but not i‐IGT [32]. The inconsistent finding for i‐IGT may reflect methodological differences, as the previous study estimated fat mass using BMI‐based prediction equations and adjusted for a broader range of risk factors, including dietary factors, anthropometric measures, lipid profiles, and blood pressure. Overall, our findings suggest that excess fat accumulation is associated with higher odds of all three prediabetes phenotypes, highlighting the value of assessing adiposity beyond BMI alone in diabetes prevention.

Higher LMI was associated with higher odds of all three prediabetes phenotypes in the main analyses. However, after additional adjustment for FMI, these associations were attenuated, and the association with i‐IGT was no longer significant, suggesting that greater total adiposity may partly explain the observed associations. Previous studies similarly found that the associations of lean mass indicators and glycemic risk vary according to whether and how fat mass is accounted for [33, 34]. A recent NHANES study also reported positive associations of both fat‐free mass index and FMI with prediabetes and diabetes, even after mutual adjustment [35]. One possible explanation is that LMI captures lean mass quantity rather than muscle quality and therefore may not adequately capture the aspects of skeletal muscle most relevant to glucose metabolism. Indeed, DEXA‐derived lean mass includes skeletal muscle as well as other nonfat soft tissues [36] and does not assess muscle composition or function, such as intramuscular lipid infiltration, fiber characteristics, oxidative capacity, or insulin‐mediated glucose disposal [35]. Future studies using more precise measures of muscle composition and function may help clarify the specific role of skeletal muscle in distinct prediabetes phenotypes.

Notably, preferential central fat deposition was associated with higher odds of i‐IFG and IFG + IGT, but not i‐IGT. This pattern may reflect the unique metabolic effects of VAT, which is closely linked to hepatic fat accumulation, inflammation, and insulin resistance [37]. Hepatic insulin resistance impairs the suppression of hepatic glucose production, a key mechanism underlying elevated FPG, but may have less influence on 2 h‐PG regulation [3, 13].

Few studies have examined the association between regional adiposity and three prediabetes phenotypes, and most have focused on absolute VAT mass or volume [14, 15]. Overall, prior evidence is broadly consistent with our findings. A cross‐sectional study of 2,515 individuals from 29 countries reported positive associations of VAT volume with i‐IFG and IFG + IGT in both sexes, but with i‐IGT only among males, and VAT volume was also higher in i‐IFG than in i‐IGT [15]. In contrast, a smaller German study (*n* = 313) reported small increases in magnetic resonance imaging‐derived VAT mass across normoglycemia, i‐IFG, i‐IGT, and IFG + IGT, after adjusting for age and sex [14]. However, this pattern may have been confounded by total adiposity, as individuals with i‐IFG had lower BMI and total body fat mass than those with i‐IGT and IFG + IGT [14].

We further extended previous work by examining peripheral adiposity. Higher proportions of total fat in appendicular or gynoid regions were associated with lower odds of i‐IFG and IFG + IGT, possibly reflecting the capacity of peripheral fat storage to buffer against central fat accumulation. However, only a higher proportion of gynoid fat was associated with lower odds of i‐IGT, supporting a potential protective role of gynoid fat distribution. In contrast, appendicular fat may partly reflect fat surrounding or infiltrating skeletal muscle, which may contribute to muscle insulin resistance and elevated 2 h‐PG [3, 10]. This finding further highlights the potential importance of skeletal muscle quality and function in postprandial glucose regulation.

The detailed DEXA assessment of general and regional adiposity provides mechanistic insight into phenotype‐specific adiposity patterns, which may inform tailored diabetes prevention strategies. However, because DEXA is not routinely available in clinical or community settings, we also examined WC as a more accessible indicator of central adiposity. WC showed a phenotype‐specific pattern broadly consistent with DEXA‐derived abdominal and visceral adiposity: elevated WC was associated with higher odds of all three prediabetes phenotypes, with stronger associations for i‐IFG and IFG + IGT. Similarly, a previous NHANES analysis found that WC was higher among individuals with i‐IFG and IFG + IGT, but not i‐IGT, than among those with normoglycemia [32]. These findings suggest that WC may partly capture the central fat distribution characteristic of i‐IFG and IFG + IGT, although it cannot distinguish fat mass from lean mass or visceral from subcutaneous abdominal fat. Overall, reducing central adiposity may be particularly relevant for i‐IFG and IFG + IGT, and WC may serve as a practical measure for monitoring central adiposity in real‐world lifestyle interventions.

Additional adjustment for HOMA‐IR attenuated most associations between adiposity indicators and prediabetes phenotypes, suggesting that insulin resistance may partly mediate adiposity‐related glycemic deterioration. This interpretation is consistent with previous prospective evidence showing that associations of visceral and subcutaneous adiposity with incident Type 2 diabetes were weakened after accounting for insulin resistance [38]. Notably, the attenuation was most pronounced for i‐IFG, which may reflect the fact that HOMA‐IR is derived from fasting glucose and insulin and therefore primarily captures fasting‐state insulin resistance, particularly hepatic insulin resistance, rather than peripheral glucose disposal [39].

Previous meta‐analyses suggest that lifestyle interventions may be less effective in reducing Type 2 diabetes incidence among individuals with i‐IFG [6, 7]. Our findings suggest that this limited effectiveness may partly reflect inadequate targeting of central adiposity, particularly VAT, and interventions specifically reducing central fat may have greater potential to improve FPG regulation [40, 41]. Given that body fat distribution is partly genetically determined [42], further studies are needed to clarify how genetic factors, body fat distribution, and prediabetes phenotypes interact, and whether genetic susceptibility to central fat accumulation contributes to elevated FPG.

The strengths of our study include the use of a nationally representative sample, both conventional anthropometric and DEXA‐derived body composition measures, and comprehensive analyses, including non‐linearity analyses, stratified analyses to examine the consistency of findings, and exploratory analyses to provide mechanistic insights. This study also has some limitations. First, the cross‐sectional design precludes causal inference. Second, our study sample consisted mainly of Hispanic and non‐Hispanic White individuals, which may limit generalizability to other ethnic groups [43]. Third, because DEXA data were available only for individuals aged < 60 years, our findings may not apply to older adults. However, this age restriction may also be a methodological strength, as it may have reduced confounding from age‐related multimorbidity, sarcopenia, and polypharmacy [44]. Fourth, DEXA‐derived VAT is an algorithm‐based estimate and may be subject to measurement imprecision, including limited differentiation between visceral and subcutaneous abdominal fat and analyst‐dependent variability in reference line placement [45].

## 5. Conclusions

Obesity, defined by BMI, and elevated general adiposity, measured by FMI, were associated with higher odds of all three prediabetes phenotypes. Of note, BMI showed a U‐shaped association with i‐IGT. Central fat distribution was associated with higher odds of i‐IFG and IFG + IGT. These findings highlight the importance of obesity management for reducing prediabetes and subsequent Type 2 diabetes risk. In addition, skeletal muscle health may be particularly relevant to postprandial glucose regulation, whereas reducing central adiposity, particularly visceral fat, may be especially important for FPG regulation.

## Author Contributions

**Jincheng Rong**: conceptualization, methodology, formal analysis, investigation, writing – original draft, writing – review and editing. **Mandy Ho**: methodology, supervision, writing – review and editing. **Sarah Garnett**: methodology, writing – review and editing. **Pui Hing Chau**: methodology, writing – review and editing.

## Funding

This study was supported by University of Hong Kong, 10.13039/501100003803.

## Ethics Statement

NHANES has been approved by the National Center for Health Statistics Research Ethics Review Board.

## Consent

Informed consent was obtained from all individual participants included in the study.

## Conflicts of Interest

The authors declare no conflicts of interest.

## Supporting information


**Supporting Information** Additional supporting information can be found online in the Supporting Information section. Table S1: Weight status, general adiposity, total lean mass, and body fat distribution indicators. Table S2: Exploratory analysis by further adjusting for HOMA‐IR. Table S3: Exploratory analysis by further adjusting for FMI in the LMI models. Table S4: Stratified analyses by sex. Table S5: Stratified analyses by age group. Table S6: Stratified analyses by ethnicity. Table S7: Stratified analyses by weight status. Figure S1: Flowchart of the study population selection process. Figure S2: Restricted cubic spline analyses.

## Data Availability

The data that support the findings of this study are openly available in National Center for Health Statistics at https://www.cdc.gov/nchs/nhanes/.
