# Supplementary material for: Prediabetes Phenotypes and Adiposity Patterns: Findings From a Population‐Based Study
Source: J Diabetes Res. 2026 Jul 20;2026:1147673. doi: 10.1155/jdr/1147673 (PMC13383241; doi:10.1155/jdr/1147673)
Supplement: Supplementary file 1 — Supporting Information Additional supporting information can be found online in the Supporting Information section. Table S1: Weight status, general adiposity, total lean mass, and body fat distribution indicators. Table S2: Exploratory analysis by further adjusting for HOMA‐IR. Table S3: Exploratory analysis by further adjusting for FMI in the LMI models. Table S4: Stratified analyses by sex. Table S5: Stratified analyses by age group. Table S6: Stratified analyses by ethnicity. Table S7: Stratified analyses by weight status. Figure S1: Flowchart of the study population selection process. Figure S2: Restricted cubic spline analyses. [file JDR-2026-1147673-s001.docx]

Title: Pre-Diabetes Phenotypes and Adiposity Patterns: Findings from a Population-based Study

Table of Contents

[Supplementary Table 1 Weight status, general adiposity, lean mass, and body fat distribution indicators 2](#_Toc232538344)

[Supplementary Table 2 Exploratory analysis by further adjusting for HOMA-IR 3](#_Toc232538345)

[Supplementary Table 3 Exploratory analysis by further adjusting for FMI in the LMI models 4](#_Toc232538346)

[Supplementary Tables 4 Stratified Analyses by sex 5](#_Toc232538347)

[Supplementary Tables 5 Stratified analyses by age group 8](#_Toc232538348)

[Supplementary Tables 6 Stratified analyses by ethnicity 11](#_Toc232538349)

[Supplementary Tables 7 Stratified analyses by weight status 14](#_Toc232538350)

[Supplementary Figure 1 Flowchart of the study population selection process 17](#_Toc232538351)

[Supplementary Figure 2 Restricted cubic spline analyses 18](#_Toc232538352)

# Supplementary Table 1 Weight status, general adiposity, lean mass, and body fat distribution indicators

**Table S1** Detailed information on weight status, general adiposity, total lean mass, and body fat distribution indicators

| **Categories** | **Indicator** | **Formula** |
| --- | --- | --- |
| **Weight Status** | BMI (kg/m^2^) | $\frac{\boldsymbol{Total body weight (kg)}}{\boldsymbol{Height square (}\boldsymbol{m}^{\boldsymbol{2}}\boldsymbol{)}}$ |
| **General Adiposity** | FMI (kg/m^2^) | $\frac{\boldsymbol{Total body fat mass (kg)}}{\boldsymbol{Height square (}\boldsymbol{m}^{\boldsymbol{2}}\boldsymbol{)}}$ |
| **Total Lean Mass** | LMI (kg/m^2^) | $\frac{\boldsymbol{Total lean mass (kg)}}{\boldsymbol{Height square (}\boldsymbol{m}^{\boldsymbol{2}}\boldsymbol{)}}$ |
| **Body Fat Distribution** | WC (cm) | Not applicable |
|  | Appendicular Adipose Tissue (%) | $\frac{\boldsymbol{Arm fat mass}\left( \boldsymbol{kg} \right)\boldsymbol{+Leg fat mass (kg)}}{\boldsymbol{Total body fat mass (kg)}}$ $\boldsymbol{\times}$ 100% |
|  | ^*^Gynoid Adipose Tissue (%) | $\frac{\boldsymbol{Gynoid fat mass (kg)}}{\boldsymbol{Total body fat mass (kg)}}$ $\times$ 100% |
|  | ^†^Abdominal Adipose Tissue (%) | $\frac{\boldsymbol{Abdominal fat mass (kg)}}{\boldsymbol{Total body fat mass (kg)}}$ $\boldsymbol{\times}$ 100% |
|  | ^†^Visceral Adipose Tissue (%) | $\frac{\boldsymbol{Visceral fat mass (kg)}}{\boldsymbol{Total body fat mass (kg)}}$ $\boldsymbol{\times}$ 100% |

**Notes:** BMI=body mass index; FMI=fat mass index; LMI=lean mass index; WC=waist circumference.

^*^Android region was measured from the pelvis cut (lower boundary) to the line above the pelvis cut by 20% of the distance between the pelvis and neck cuts (upper boundary). Gynoid region was measured from the line below the pelvis cut by 1.5 times the height of the android region (upper boundary), and the height was equal to twice the height of the android region.

^†^ The abdomen region is defined by the Hologic APEX software used in the scan analysis, as measured at the approximate interspace location of L4 and L5 vertebra. Abdominal adipose tissue contains both visceral and subcutaneous adipose tissue. The software detected the fat located on both sides (inside and outside) of the abdominal cavity. Visceral adipose tissue was defined as fat inside the abdominal cavity.

# Supplementary Table 2 Exploratory analysis by further adjusting for HOMA-IR

**Table S2** Exploratory analysis by further adjusting for HOMA-IR

|  | **I-IFG** | | | **I-IGT** | | | **IFG+IGT** | | |
| --- | --- | --- | --- | --- | --- | --- | --- | --- | --- |
|  | **OR** | **95% CI** | **p value** | **OR** | **95% CI** | **p value** | **OR** | **95% CI** | **p value** |
| **Weight Status** |  |  |  |  |  |  |  |  |  |
| **BMI kg/m^2^** |  |  |  |  |  |  |  |  |  |
| Normal | Ref |  |  | Ref |  |  | Ref |  |  |
| Underweight | 0.70 | 0.27,1.77 | 0.433 | 3.40 | 1.26,9.15 | 0.017 | 1.06 | 0.27,4.11 | 0.934 |
| Overweight | 1.16 | 0.89,1.51 | 0.269 | 0.90 | 0.49,1.65 | 0.729 | 2.02 | 1.12,3.67 | 0.022 |
| Obesity | 1.17 | 0.85,1.61 | 0.313 | 1.97 | 1.22,3.18 | 0.007 | 2.23 | 1.17,4.23 | 0.016 |
| **General Adiposity** |  |  |  |  |  |  |  |  |  |
| **FMI, kg/m^2^** |  |  |  |  |  |  |  |  |  |
| Tertile 1 | Ref |  |  | Ref |  |  | Ref |  |  |
| Tertile 2 | 1.13 | 0.86,1.48 | 0.381 | 0.84 | 0.50,1.40 | 0.486 | 2.73 | 1.40,5.33 | 0.004 |
| Tertile 3 | 1.23 | 0.83,1.83 | 0.297 | 1.56 | 0.95,2.55 | 0.077 | 3.26 | 1.39,7.60 | 0.008 |
| **Total Lean Mass** |  |  |  |  |  |  |  |  |  |
| **LMI, kg/m^2^** |  |  |  |  |  |  |  |  |  |
| Tertile 1 | Ref |  |  | Ref |  |  | Ref |  |  |
| Tertile 2 | 1.09 | 0.82,1.44 | 0.558 | 0.90 | 0.48,1.68 | 0.721 | 1.48 | 0.84,2.62 | 0.167 |
| Tertile 3 | 1.23 | 0.90,1.67 | 0.191 | 1.30 | 0.73,2.33 | 0.362 | 2.70 | 1.57,4.64 | 0.001 |
| **Body Fat Distribution** |  |  |  |  |  |  |  |  |  |
| **WC, cm** |  |  |  |  |  |  |  |  |  |
| Normal | Ref |  |  | Ref |  |  | Ref |  |  |
| High-risk central obesity | 1.35 | 1.01,1.80 | 0.043 | 0.93 | 0.47,1.83 | 0.827 | 2.25 | 1.25,4.04 | 0.008 |
| Very-high-risk central obesity | 1.30 | 0.88,1.90 | 0.181 | 1.34 | 0.79,2.28 | 0.268 | 3.66 | 1.94,6.89 | <0.001 |
| ^*^**Appendicular adipose tissue, %** |  |  |  |  |  |  |  |  |  |
| Tertile 1 | Ref |  |  | Ref |  |  | Ref |  |  |
| Tertile 2 | 0.70 | 0.52,0.94 | 0.021 | 1.06 | 0.64,1.74 | 0.823 | 0.49 | 0.30,0.79 | 0.005 |
| Tertile 3 | 0.67 | 0.49,0.91 | 0.012 | 0.96 | 0.57,1.62 | 0.875 | 0.52 | 0.29,0.93 | 0.029 |
| ^*^**Gynoid adipose tissue, %** |  |  |  |  |  |  |  |  |  |
| Tertile 1 | Ref |  |  | Ref |  |  | Ref |  |  |
| Tertile 2 | 0.85 | 0.63,1.17 | 0.309 | 0.86 | 0.56,1.32 | 0.477 | 0.57 | 0.33,0.96 | 0.037 |
| Tertile 3 | 0.84 | 0.59,1.19 | 0.314 | 0.54 | 0.31,0.95 | 0.033 | 0.65 | 0.37,1.16 | 0.138 |
| ^*^**Abdominal adipose tissue, %** |  |  |  |  |  |  |  |  |  |
| Tertile 1 | Ref |  |  | Ref |  |  | Ref |  |  |
| Tertile 2 | 1.09 | 0.82,1.47 | 0.537 | 1.12 | 0.65,1.93 | 0.665 | 1.23 | 0.66,2.32 | 0.500 |
| Tertile 3 | 1.26 | 0.97,1.66 | 0.086 | 1.01 | 0.61,1.68 | 0.962 | 2.02 | 1.06,3.85 | 0.033 |
| ^*^**Visceral adipose tissue, %** |  |  |  |  |  |  |  |  |  |
| Tertile 1 | Ref |  |  | Ref |  |  | Ref |  |  |
| Tertile 2 | 1.30 | 1.00,1.70 | 0.049 | 1.38 | 0.74,2.58 | 0.293 | 1.74 | 0.88,3.47 | 0.109 |
| Tertile 3 | 1.27 | 0.95,1.68 | 0.100 | 1.19 | 0.68,2.08 | 0.534 | 2.62 | 1.39,4.96 | 0.004 |

**Note:** BMI=body mass index; CI=confidence interval; FMI=fat mass index; HOMA-IR=Homeostatic Model Assessment for Insulin Resistance; LMI=lean mass index; I-IFG=isolated-impaired fasting glucose; IFG+IGT=impaired both fasting glucose and glucose tolerance; I-IGT=isolated-impaired glucose tolerance; OR=odds ratio; Ref=reference; WC=waist circumference.

All analyses were weighted and accounted for the complex survey design. Models were adjusted for age, sex, ethnicity, education, poverty income ratio, physical activity, smoking, alcohol drinking, family history of diabetes, and HOMA-IR. Models for body fat distribution variables were additionally adjusted for height.

^*^ DEXA-derived body fat distribution variables were expressed as regional fat mass relative to total body fat mass, including appendicular adipose tissue (%), gynoid adipose tissue (%), abdominal adipose tissue (%) and visceral adipose tissue (%).

# Supplementary Table 3 Exploratory analysis by further adjusting for FMI in the LMI models

**Table S3** Exploratory analysis by further adjusting for FMI in the models for LMI

|  | **I-IFG** | | | **I-IGT** | | | **IFG+IGT** | | |
| --- | --- | --- | --- | --- | --- | --- | --- | --- | --- |
|  | **OR** | **95% CI** | **p value** | **OR** | **95% CI** | **p value** | **OR** | **95% CI** | **p value** |
| **LMI, kg/m^2^** |  |  |  |  |  |  |  |  |  |
| Tertile 1 | Ref |  |  | Ref |  |  | Ref |  |  |
| Tertile 2 | 1.17 | 0.88, 1.55 | 0.270 | 0.91 | 0.48, 1.72 | 0.773 | 1.59 | 0.93, 2.72 | 0.089 |
| Tertile 3 | 1.51 | 1.01, 2.24 | 0.044 | 1.34 | 0.68, 2.64 | 0.378 | 4.08 | 2.43, 6.85 | <0.001 |

**Note:** CI=confidence interval; FMI=fat mass index; LMI=lean mass index; I-IFG=isolated-impaired fasting glucose; IFG+IGT=impaired both fasting glucose and glucose tolerance; I-IGT=isolated-impaired glucose tolerance; OR=odds ratio; Ref=reference; WC=waist circumference.

All analyses were weighted and accounted for the complex survey design. Models were adjusted for age, sex, ethnicity, education, poverty income ratio, physical activity, smoking, alcohol drinking, family history of diabetes, and FMI.

# Supplementary Tables 4 Stratified Analyses by sex

**Table S4.1** Logistic regression models for the associations of weight status, general adiposity, total lean mass, and body fat distribution with I-IFG by sex

|  | **I-IFG** |  |  |  |  |  |  |  |
| --- | --- | --- | --- | --- | --- | --- | --- | --- |
|  | **Female** |  |  |  | **Male** |  |  |  |
|  | **OR** | **95% CI** | **p value** |  | **OR** | **95% CI** | **p value** | **p interaction** |
| **Weight Status** |  |  |  |  |  |  |  |  |
| **BMI kg/m^2^** |  |  |  |  |  |  |  | 0.409 |
| Normal | Ref |  |  |  | Ref |  |  |  |
| Underweight | 0.89 | 0.19, 4.27 | 0.885 |  | 0.33 | 0.11, 1.01 | 0.053 |  |
| Overweight | 2.11 | 1.26, 3.51 | 0.006 |  | 1.34 | 0.92, 1.94 | 0.124 |  |
| Obesity | 3.48 | 2.12, 5.72 | <0.001 |  | 2.35 | 1.62, 3.41 | <0.001 |  |
| **General Adiposity** |  |  |  |  |  |  |  |  |
| **FMI, kg/m^2^** |  |  |  |  |  |  |  | 0.186 |
| Tertile 1 | Ref |  |  |  | Ref |  |  |  |
| Tertile 2 | 2.09 | 1.25, 3.50 | 0.007 |  | 1.35 | 0.96, 1.88 | 0.081 |  |
| Tertile 3 | 3.87 | 2.20, 6.80 | <0.001 |  | 2.40 | 1.68, 3.43 | <0.001 |  |
| **Total Lean Mass** |  |  |  |  |  |  |  |  |
| **LMI, kg/m^2^** |  |  |  |  |  |  |  | 0.174 |
| Tertile 1 | Ref |  |  |  | Ref |  |  |  |
| Tertile 2 | 1.86 | 1.15, 3.00 | 0.013 |  | 1.20 | 0.81, 1.76 | 0.352 |  |
| Tertile 3 | 3.94 | 2.40, 6.48 | <0.001 |  | 2.04 | 1.45, 2.88 | <0.001 |  |
| **Body Fat Distribution** |  |  |  |  |  |  |  |  |
| **WC, cm** |  |  |  |  |  |  |  | 0.090 |
| Normal | Ref |  |  |  | Ref |  |  |  |
| High-risk central obesity | 1.83 | 0.88, 3.82 | 0.102 |  | 2.17 | 1.56, 3.02 | <0.001 |  |
| Very-high-risk central obesity | 3.65 | 1.83, 7.25 | <0.001 |  | 2.58 | 1.84, 3.62 | <0.001 |  |
| ^*^**Appendicular adipose tissue, %** |  |  |  |  |  |  |  | 0.410 |
| Tertile 1 | Ref |  |  |  | Ref |  |  |  |
| Tertile 2 | 0.50 | 0.33, 0.73 | <0.001 |  | 0.60 | 0.41, 0.88 | 0.010 |  |
| Tertile 3 | 0.35 | 0.23, 0.55 | <0.001 |  | 0.42 | 0.28, 0.63 | <0.001 |  |
| ^*^**Gynoid adipose tissue, %** |  |  |  |  |  |  |  | 0.152 |
| Tertile 1 | Ref |  |  |  | Ref |  |  |  |
| Tertile 2 | 0.61 | 0.38, 0.96 | 0.035 |  | 0.61 | 0.45, 0.82 | 0.002 |  |
| Tertile 3 | 0.37 | 0.23, 0.57 | <0.001 |  | 0.48 | 0.33, 0.68 | <0.001 |  |
| ^*^**Abdominal adipose tissue, %** |  |  |  |  |  |  |  | 0.082 |
| Tertile 1 | Ref |  |  |  | Ref |  |  |  |
| Tertile 2 | 1.06 | 0.71, 1.58 | 0.780 |  | 2.20 | 1.54, 3.14 | <0.001 |  |
| Tertile 3 | 1.58 | 1.01, 2.48 | 0.045 |  | 2.78 | 1.86, 4.15 | <0.001 |  |
| ^*^**Visceral adipose tissue, %** |  |  |  |  |  |  |  | 0.018 |
| Tertile 1 | Ref |  |  |  | Ref |  |  |  |
| Tertile 2 | 2.44 | 1.41, 4.22 | 0.002 |  | 0.99 | 0.69, 1.41 | 0.934 |  |
| Tertile 3 | 2.45 | 1.34, 4.46 | 0.005 |  | 1.37 | 0.98, 1.91 | 0.068 |  |

**Note:** BMI=body mass index; CI=confidence interval; FMI=fat mass index; LMI=lean mass index; I-IFG=isolated-impaired fasting glucose; OR=odds ratio; Ref=reference; WC=waist circumference.

All analyses were weighted and accounted for the complex survey design. Models were adjusted for age, ethnicity, education, poverty income ratio, physical activity, smoking, alcohol drinking, and family history of diabetes. Models for fat distribution variables were additionally adjusted for height.

^*^ DEXA-derived body fat distribution variables were expressed as regional fat mass relative to total body fat mass, including appendicular adipose tissue (%), gynoid adipose tissue (%), abdominal adipose tissue (%) and visceral adipose tissue (%).

**Table S4.2** Logistic regression models for the associations of weight status, general adiposity, total lean mass, and body fat distribution with I-IGT by sex

|  | **I-IGT** |  |  |  |  |  |  |  |
| --- | --- | --- | --- | --- | --- | --- | --- | --- |
|  | **Female** |  |  |  | **Male** |  |  |  |
|  | **OR** | **95% CI** | **p value** |  | **OR** | **95% CI** | **p value** | **p interaction** |
| **Weight Status** |  |  |  |  |  |  |  |  |
| **BMI kg/m^2^** |  |  |  |  |  |  |  | 0.935 |
| Normal | Ref |  |  |  | Ref |  |  |  |
| Underweight | 3.31 | 1.33, 8.23 | 0.012 |  | 4.89 | 0.57, 42.36 | 0.144 |  |
| Overweight | 1.10 | 0.48, 2.54 | 0.821 |  | 1.11 | 0.39, 3.21 | 0.837 |  |
| Obesity | 2.53 | 1.29, 4.96 | 0.009 |  | 4.01 | 1.73, 9.28 | 0.002 |  |
| **General Adiposity** |  |  |  |  |  |  |  |  |
| **FMI, kg/m^2^** |  |  |  |  |  |  |  | 0.809 |
| Tertile 1 | Ref |  |  |  | Ref |  |  |  |
| Tertile 2 | 1.04 | 0.49, 2.24 | 0.911 |  | 0.87 | 0.29, 2.58 | 0.790 |  |
| Tertile 3 | 2.04 | 1.05, 3.98 | 0.037 |  | 2.72 | 1.09, 6.78 | 0.033 |  |
| **Total Lean Mass** |  |  |  |  |  |  |  |  |
| **LMI, kg/m^2^** |  |  |  |  |  |  |  | 0.981 |
| Tertile 1 | Ref |  |  |  | Ref |  |  |  |
| Tertile 2 | 0.96 | 0.38, 2.39 | 0.927 |  | 1.17 | 0.49, 2.80 | 0.709 |  |
| Tertile 3 | 1.71 | 0.79, 3.68 | 0.166 |  | 2.36 | 0.99, 5.60 | 0.052 |  |
| **Body Fat Distribution** |  |  |  |  |  |  |  |  |
| **WC, cm** |  |  |  |  |  |  |  | 0.538 |
| Normal | Ref |  |  |  | Ref |  |  |  |
| High-risk central obesity | 0.85 | 0.30, 2.37 | 0.749 |  | 1.45 | 0.53, 3.96 | 0.461 |  |
| Very-high-risk central obesity | 1.52 | 0.75, 3.11 | 0.239 |  | 2.91 | 1.22, 6.95 | 0.018 |  |
| ^*^**Appendicular adipose tissue, %** |  |  |  |  |  |  |  | 0.981 |
| Tertile 1 | Ref |  |  |  | Ref |  |  |  |
| Tertile 2 | 0.84 | 0.47, 1.50 | 0.547 |  | 1.00 | 0.40, 2.50 | 0.997 |  |
| Tertile 3 | 0.69 | 0.29, 1.63 | 0.385 |  | 0.72 | 0.29, 1.78 | 0.465 |  |
| ^*^**Gynoid adipose tissue, %** |  |  |  |  |  |  |  | 0.107 |
| Tertile 1 | Ref |  |  |  | Ref |  |  |  |
| Tertile 2 | 0.96 | 0.53, 1.72 | 0.875 |  | 0.41 | 0.18, 0.92 | 0.032 |  |
| Tertile 3 | 0.36 | 0.17, 0.79 | 0.012 |  | 0.45 | 0.23, 0.88 | 0.021 |  |
| ^*^**Abdominal adipose tissue, %** |  |  |  |  |  |  |  | 0.915 |
| Tertile 1 | Ref |  |  |  | Ref |  |  |  |
| Tertile 2 | 1.37 | 0.70, 2.69 | 0.348 |  | 1.54 | 0.64, 3.69 | 0.321 |  |
| Tertile 3 | 1.49 | 0.70, 3.18 | 0.289 |  | 1.34 | 0.63, 2.86 | 0.434 |  |
| ^*^**Visceral adipose tissue, %** |  |  |  |  |  |  |  | 0.030 |
| Tertile 1 | Ref |  |  |  | Ref |  |  |  |
| Tertile 2 | 2.46 | 1.24, 4.88 | 0.012 |  | 0.60 | 0.22, 1.59 | 0.290 |  |
| Tertile 3 | 1.66 | 0.83, 3.29 | 0.144 |  | 1.03 | 0.42, 2.52 | 0.944 |  |

**Note:** BMI=body mass index; CI=confidence interval; FMI=fat mass index; LMI=lean mass index; I-IGT=isolated-impaired glucose tolerance; OR=odds ratio; Ref=reference; WC=waist circumference.

All analyses were weighted and accounted for the complex survey design. Models were adjusted for age, ethnicity, education, poverty income ratio, physical activity, smoking, alcohol drinking, and family history of diabetes. Models for fat distribution variables were additionally adjusted for height.

^*^ DEXA-derived body fat distribution variables were expressed as regional fat mass relative to total body fat mass, including appendicular adipose tissue (%), gynoid adipose tissue (%), abdominal adipose tissue (%) and visceral adipose tissue (%).

**Table S4.3** Logistic regression models for the associations of weight status, general adiposity, total lean mass, and body fat distribution with IFG+IGT by sex

|  | **IFG+IGT** |  |  |  |  |  |  |  |
| --- | --- | --- | --- | --- | --- | --- | --- | --- |
|  | **Female** |  |  |  | **Male** |  |  |  |
|  | **OR** | **95% CI** | **p value** |  | **OR** | **95% CI** | **p value** | **p interaction** |
| **Weight Status** |  |  |  |  |  |  |  |  |
| **BMI kg/m^2^** |  |  |  |  |  |  |  | 0.644 |
| Normal | Ref |  |  |  | Ref |  |  |  |
| Underweight | 0.40 | 0.05, 3.34 | 0.382 |  | 1.43 | 0.25, 8.30 | 0.683 |  |
| Overweight | 4.10 | 1.79, 9.40 | 0.002 |  | 2.38 | 1.20, 4.72 | 0.015 |  |
| Obesity | 8.14 | 3.78, 17.52 | <0.001 |  | 6.36 | 3.08, 13.11 | <0.001 |  |
| **General Adiposity** |  |  |  |  |  |  |  |  |
| **FMI, kg/m^2^** |  |  |  |  |  |  |  | 0.801 |
| Tertile 1 | Ref |  |  |  | Ref |  |  |  |
| Tertile 2 | 4.68 | 1.98, 11.08 | <0.001 |  | 4.18 | 1.87, 9.34 | <0.001 |  |
| Tertile 3 | 9.47 | 3.52, 25.52 | <0.001 |  | 11.01 | 5.06, 23.94 | <0.001 |  |
| **Total Lean Mass** |  |  |  |  |  |  |  |  |
| **LMI, kg/m^2^** |  |  |  |  |  |  |  | 0.198 |
| Tertile 1 | Ref |  |  |  | Ref |  |  |  |
| Tertile 2 | 3.23 | 1.47, 7.11 | 0.005 |  | 1.31 | 0.66, 2.62 | 0.428 |  |
| Tertile 3 | 9.24 | 4.56, 18.72 | <0.001 |  | 6.09 | 3.23, 11.47 | <0.001 |  |
| **Body Fat Distribution** |  |  |  |  |  |  |  |  |
| **WC, cm** |  |  |  |  |  |  |  | 0.255 |
| Normal | Ref |  |  |  | Ref |  |  |  |
| High-risk central obesity | 3.32 | 1.04, 10.61 | 0.044 |  | 4.56 | 2.04, 10.22 | <0.001 |  |
| Very-high-risk central obesity | 14.67 | 6.50, 33.14 | <0.001 |  | 11.20 | 5.54, 22.66 | <0.001 |  |
| ^*^**Appendicular adipose tissue, %** |  |  |  |  |  |  |  | 0.335 |
| Tertile 1 | Ref |  |  |  | Ref |  |  |  |
| Tertile 2 | 0.48 | 0.27, 0.84 | 0.012 |  | 0.22 | 0.11, 0.43 | <0.001 |  |
| Tertile 3 | 0.34 | 0.14, 0.82 | 0.019 |  | 0.16 | 0.07, 0.36 | <0.001 |  |
| ^*^**Gynoid adipose tissue, %** |  |  |  |  |  |  |  | 0.338 |
| Tertile 1 | Ref |  |  |  | Ref |  |  |  |
| Tertile 2 | 0.44 | 0.22, 0.87 | 0.019 |  | 0.23 | 0.14, 0.37 | <0.001 |  |
| Tertile 3 | 0.21 | 0.09, 0.52 | 0.001 |  | 0.25 | 0.12, 0.51 | <0.001 |  |
| ^*^**Abdominal adipose tissue, %** |  |  |  |  |  |  |  | 0.222 |
| Tertile 1 | Ref |  |  |  | Ref |  |  |  |
| Tertile 2 | 1.43 | 0.62, 3.30 | 0.388 |  | 3.73 | 1.81, 7.68 | <0.001 |  |
| Tertile 3 | 2.96 | 1.23, 7.12 | 0.017 |  | 7.00 | 3.53, 13.90 | <0.001 |  |
| ^*^**Visceral adipose tissue, %** |  |  |  |  |  |  |  | 0.302 |
| Tertile 1 | Ref |  |  |  | Ref |  |  |  |
| Tertile 2 | 2.53 | 0.74, 8.68 | 0.135 |  | 1.16 | 0.50, 2.72 | 0.719 |  |
| Tertile 3 | 6.51 | 2.06, 20.54 | 0.002 |  | 1.93 | 0.85, 4.37 | 0.113 |  |

**Note:** BMI=body mass index; CI=confidence interval; IFG+IGT=impaired both fasting plasma glucose and glucose tolerance; FMI=fat mass index; LMI=lean mass index; OR=odds ratio; Ref=reference; WC=waist circumference.

All analyses were weighted and accounted for the complex survey design. Models were adjusted for age, ethnicity, education, poverty income ratio, physical activity, smoking, alcohol drinking, and family history of diabetes. Models for fat distribution variables were additionally adjusted for height.

^*^ DEXA-derived body fat distribution variables were expressed as regional fat mass relative to total body fat mass, including appendicular adipose tissue (%), gynoid adipose tissue (%), abdominal adipose tissue (%) and visceral adipose tissue (%).

# Supplementary Tables 5 Stratified analyses by age group

**Table S5.1** Logistic regression models for the associations of weight status, general adiposity, total lean mass, and body fat distribution with I-IFG by age group

|  | **I-IFG** |  |  |  |  |  |  |  |
| --- | --- | --- | --- | --- | --- | --- | --- | --- |
|  | **Age Group 18-44 years** | | |  | **Age Group 45-59 years** | | |  |
|  | **OR** | **95% CI** | **p value** |  | **OR** | **95% CI** | **p value** | **p interaction** |
| **Weight Status** |  |  |  |  |  |  |  |  |
| **BMI kg/m^2^** |  |  |  |  |  |  |  | 0.705 |
| Normal | Ref |  |  |  | Ref |  |  |  |
| Underweight | 0.70 | 0.23, 2.13 | 0.514 |  | 0.21 | 0.03, 1.33 | 0.094 |  |
| Overweight | 1.59 | 1.15, 2.21 | 0.007 |  | 1.53 | 0.93, 2.52 | 0.093 |  |
| Obesity | 2.81 | 2.12, 3.74 | <0.001 |  | 2.74 | 1.55, 4.85 | 0.001 |  |
| **General Adiposity** |  |  |  |  |  |  |  |  |
| **FMI, kg/m^2^** |  |  |  |  |  |  |  | 0.598 |
| Tertile 1 | Ref |  |  |  |  | Ref |  |  |
| Tertile 2 | 1.51 | 1.13, 2.02 | 0.007 |  | 1.81 | 1.10, 2.97 | 0.020 |  |
| Tertile 3 | 2.71 | 2.05, 3.59 | <0.001 |  | 3.49 | 1.97, 6.17 | <0.001 |  |
| **Total Lean Mass** |  |  |  |  |  |  |  |  |
| **LMI, kg/m^2^** |  |  |  |  |  |  |  | 0.669 |
| Tertile 1 | Ref |  |  |  |  | Ref |  |  |
| Tertile 2 | 1.29 | 0.86, 1.94 | 0.217 |  | 1.76 | 1.14, 2.73 | 0.013 |  |
| Tertile 3 | 2.57 | 1.94, 3.40 | <0.001 |  | 2.92 | 1.50, 5.71 | 0.003 |  |
| **Body Fat Distribution** |  |  |  |  |  |  |  |  |
| **WC, cm** |  |  |  |  |  |  |  | 0.348 |
| Normal | Ref |  |  |  |  | Ref |  |  |
| High-risk central obesity | 2.22 | 1.64, 3.01 | <0.001 |  | 1.38 | 0.70, 2.72 | 0.345 |  |
| Very-high-risk central obesity | 3.17 | 2.42, 4.14 | <0.001 |  | 2.45 | 1.22, 4.91 | 0.013 |  |
| ^*^**Appendicular adipose tissue, %** |  |  |  |  |  |  |  | 0.317 |
| Tertile 1 | Ref |  |  |  |  | Ref |  |  |
| Tertile 2 | 0.60 | 0.43, 0.84 | 0.004 |  | 0.47 | 0.27, 0.82 | 0.009 |  |
| Tertile 3 | 0.37 | 0.26, 0.52 | <0.001 |  | 0.46 | 0.26, 0.79 | 0.006 |  |
| ^*^**Gynoid adipose tissue, %** |  |  |  |  |  |  |  | 0.275 |
| Tertile 1 | Ref |  |  |  |  | Ref |  |  |
| Tertile 2 | 0.63 | 0.46, 0.87 | 0.007 |  | 0.53 | 0.32, 0.86 | 0.012 |  |
| Tertile 3 | 0.39 | 0.28, 0.55 | <0.001 |  | 0.53 | 0.30, 0.91 | 0.023 |  |
| ^*^**Abdominal adipose tissue, %** |  |  |  |  |  |  |  | 0.853 |
| Tertile 1 | Ref |  |  |  |  | Ref |  |  |
| Tertile 2 | 1.71 | 1.19, 2.47 | 0.006 |  | 1.53 | 0.92, 2.57 | 0.101 |  |
| Tertile 3 | 2.41 | 1.59, 3.64 | <0.001 |  | 1.95 | 1.14, 3.34 | 0.017 |  |
| ^*^**Visceral adipose tissue, %** |  |  |  |  |  |  |  | 0.085 |
| Tertile 1 | Ref |  |  |  |  | Ref |  |  |
| Tertile 2 | 1.20 | 0.91, 1.58 | 0.196 |  | 2.38 | 1.20, 4.71 | 0.015 |  |
| Tertile 3 | 1.82 | 1.32, 2.52 | <0.001 |  | 2.02 | 1.09, 3.72 | 0.026 |  |

**Note:** BMI=body mass index; CI=confidence interval; FMI=fat mass index; LMI=lean mass index; I-IFG=isolated-impaired fasting glucose; OR=odds ratio; Ref=reference; WC=waist circumference.

All analyses were weighted and accounted for the complex survey design. Models were adjusted for sex, ethnicity, education, poverty income ratio, physical activity, smoking, alcohol drinking, and family history of diabetes. Models for fat distribution variables were additionally adjusted for height.

^*^ DEXA-derived body fat distribution variables were expressed as regional fat mass relative to total body fat mass, including appendicular adipose tissue (%), abdominal adipose tissue (%), gynoid adipose tissue (%) and visceral adipose tissue (%).

**Table S5.2** Logistic regression models for the associations of weight status, general adiposity, total lean mass, and body fat distribution with I-IGT by age group

|  | **I-IGT** |  |  |  |  |  |  |  |
| --- | --- | --- | --- | --- | --- | --- | --- | --- |
|  | **Age Group 18-44 years** | | |  | **Age Group 45-59 years** | | |  |
|  | **OR** | **95% CI** | **p value** |  | **OR** | **95% CI** | **p value** | **p interaction** |
| **Weight Status** |  |  |  |  |  |  |  |  |
| **BMI kg/m^2^** |  |  |  |  |  |  |  | 0.104 |
| Normal | Ref |  |  |  | Ref |  |  |  |
| Underweight | 4.20 | 1.53, 11.53 | 0.007 |  | ^†^0.00 | 0.00, 0.00 | <0.001 |  |
| Overweight | 0.83 | 0.38, 1.81 | 0.626 |  | 1.29 | 0.55, 3.00 | 0.545 |  |
| Obesity | 2.68 | 1.51, 4.76 | 0.001 |  | 2.38 | 1.13, 5.01 | 0.024 |  |
| **General Adiposity** |  |  |  |  |  |  |  |  |
| **FMI, kg/m^2^** |  |  |  |  |  |  |  | 0.823 |
| Tertile 1 | Ref |  |  |  | Ref |  |  |  |
| Tertile 2 | 0.97 | 0.53, 1.78 | 0.929 |  | 0.86 | 0.35, 2.12 | 0.743 |  |
| Tertile 3 | 1.97 | 1.10, 3.53 | 0.025 |  | 2.34 | 1.14, 4.84 | 0.023 |  |
| **Total Lean Mass** |  |  |  |  |  |  |  |  |
| **LMI, kg/m^2^** |  |  |  |  |  |  |  | 0.467 |
| Tertile 1 | Ref |  |  |  | Ref |  |  |  |
| Tertile 2 | 0.84 | 0.34, 2.02 | 0.681 |  | 1.47 | 0.69, 3.13 | 0.312 |  |
| Tertile 3 | 1.45 | 0.74, 2.86 | 0.271 |  | 2.46 | 0.99, 6.09 | 0.052 |  |
| **Body Fat Distribution** |  |  |  |  |  |  |  |  |
| **WC, cm** |  |  |  |  |  |  |  | 0.795 |
| Normal | Ref |  |  |  | Ref |  |  |  |
| High-risk central obesity | 1.14 | 0.51, 2.58 | 0.739 |  | 1.03 | 0.32, 3.27 | 0.962 |  |
| Very-high-risk central obesity | 2.03 | 1.09, 3.76 | 0.027 |  | 1.77 | 0.72, 4.31 | 0.203 |  |
| ^*^**Appendicular adipose tissue, %** |  |  |  |  |  |  |  | 0.652 |
| Tertile 1 | Ref |  |  |  | Ref |  |  |  |
| Tertile 2 | 1.10 | 0.61, 1.98 | 0.737 |  | 0.86 | 0.36, 2.03 | 0.717 |  |
| Tertile 3 | 0.68 | 0.34, 1.38 | 0.279 |  | 1.01 | 0.37, 2.79 | 0.985 |  |
| ^*^**Gynoid adipose tissue, %** |  |  |  |  |  |  |  | 0.672 |
| Tertile 1 | Ref |  |  |  | Ref |  |  |  |
| Tertile 2 | 0.67 | 0.38, 1.18 | 0.161 |  | 0.85 | 0.39, 1.86 | 0.677 |  |
| Tertile 3 | 0.33 | 0.16, 0.65 | 0.002 |  | 0.81 | 0.36, 1.79 | 0.583 |  |
| ^*^**Abdominal adipose tissue, %** |  |  |  |  |  |  |  | 0.530 |
| Tertile 1 | Ref |  |  |  | Ref |  |  |  |
| Tertile 2 | 1.20 | 0.68, 2.12 | 0.514 |  | 2.36 | 0.67, 8.25 | 0.172 |  |
| Tertile 3 | 1.10 | 0.57, 2.15 | 0.763 |  | 1.88 | 0.61, 5.79 | 0.263 |  |
| ^*^**Visceral adipose tissue, %** |  |  |  |  |  |  |  | 0.930 |
| Tertile 1 | Ref |  |  |  | Ref |  |  |  |
| Tertile 2 | 1.40 | 0.72, 2.69 | 0.309 |  | 1.21 | 0.30, 4.89 | 0.783 |  |
| Tertile 3 | 1.60 | 0.89, 2.90 | 0.115 |  | 1.03 | 0.27, 3.92 | 0.968 |  |

**Note:** BMI=body mass index; CI=confidence interval; FMI=fat mass index; LMI=lean mass index; I-IGT=isolated-impaired glucose tolerance; OR=odds ratio; Ref=reference; WC=waist circumference.

All analyses were weighted and accounted for the complex survey design. Models were adjusted for sex, ethnicity, education, poverty income ratio, physical activity, smoking, alcohol drinking, and family history of diabetes. Models for fat distribution variables were additionally adjusted for height.

^*^ DEXA-derived body fat distribution variables were expressed as regional fat mass relative to total body fat mass, including appendicular adipose tissue (%), abdominal adipose tissue (%), gynoid adipose tissue (%) and visceral adipose tissue (%).

^†^ Estimates of 0.00 with 95% CI 0.00–0.00 indicate sparse data.

**Table S5.3** Logistic regression models for the associations of weight status, general adiposity, total lean mass, and body fat distribution with IFG+IGT by age group

|  | **IFG+IGT** |  |  |  |  |  |  |  |
| --- | --- | --- | --- | --- | --- | --- | --- | --- |
|  | **Age Group 18-44 years** | | |  | **Age Group 45-59 years** | | |  |
|  | **OR** | **95% CI** | **p value** |  | **OR** | **95% CI** | **p value** | **p interaction** |
| **Weight Status** |  |  |  |  |  |  |  |  |
| **BMI kg/m^2^** |  |  |  |  |  |  |  | 0.915 |
| Normal | Ref |  |  |  | Ref |  |  |  |
| Underweight | 0.36 | 0.04, 3.12 | 0.339 |  | 1.31 | 0.24, 7.13 | 0.744 |  |
| Overweight | 2.84 | 1.19, 6.81 | 0.021 |  | 2.83 | 1.25, 6.40 | 0.014 |  |
| Obesity | 6.47 | 3.08, 13.57 | <0.001 |  | 7.71 | 4.00, 14.88 | <0.001 |  |
| **General Adiposity** |  |  |  |  |  |  |  |  |
| **FMI, kg/m^2^** |  |  |  |  |  |  |  | 0.730 |
| Tertile 1 | Ref |  |  |  | Ref |  |  |  |
| Tertile 2 | 3.32 | 1.33, 8.28 | 0.012 |  | 6.11 | 2.56, 14.58 | <0.001 |  |
| Tertile 3 | 7.90 | 3.52, 17.73 | <0.001 |  | 13.99 | 6.44, 30.38 | <0.001 |  |
| **Total Lean Mass** |  |  |  |  |  |  |  |  |
| **LMI, kg/m^2^** |  |  |  |  |  |  |  | 0.571 |
| Tertile 1 | Ref |  |  |  | Ref |  |  |  |
| Tertile 2 | 2.63 | 1.20, 5.76 | 0.017 |  | 1.57 | 0.77, 3.20 | 0.208 |  |
| Tertile 3 | 7.74 | 4.00, 14.99 | <0.001 |  | 7.76 | 3.73, 16.17 | <0.001 |  |
| **Body Fat Distribution** |  |  |  |  |  |  |  |  |
| **WC, cm** |  |  |  |  |  |  |  | 0.849 |
| Normal | Ref |  |  |  | Ref |  |  |  |
| High-risk central obesity | 4.49 | 2.07, 9.75 | <0.001 |  | 3.69 | 1.27, 10.74 | 0.018 |  |
| Very-high-risk central obesity | 11.31 | 5.29, 24.21 | <0.001 |  | 13.08 | 5.65, 30.27 | <0.001 |  |
| ^*^**Appendicular adipose tissue, %** |  |  |  |  |  |  |  | 0.361 |
| Tertile 1 | Ref |  |  |  | Ref |  |  |  |
| Tertile 2 | 0.42 | 0.23, 0.79 | 0.008 |  | 0.24 | 0.14, 0.43 | <0.001 |  |
| Tertile 3 | 0.28 | 0.13, 0.61 | 0.002 |  | 0.19 | 0.08, 0.44 | <0.001 |  |
| ^*^**Gynoid adipose tissue, %** |  |  |  |  |  |  |  | 0.021 |
| Tertile 1 | Ref |  |  |  | Ref |  |  |  |
| Tertile 2 | 0.45 | 0.26, 0.80 | 0.008 |  | 0.21 | 0.11, 0.42 | <0.001 |  |
| Tertile 3 | 0.35 | 0.17, 0.70 | 0.004 |  | 0.08 | 0.03, 0.20 | <0.001 |  |
| ^*^**Abdominal adipose tissue, %** |  |  |  |  |  |  |  | 0.717 |
| Tertile 1 | Ref |  |  |  | Ref |  |  |  |
| Tertile 2 | 1.89 | 0.82, 4.37 | 0.133 |  | 2.81 | 1.26, 6.27 | 0.013 |  |
| Tertile 3 | 4.32 | 1.78, 10.51 | 0.002 |  | 4.51 | 2.07, 9.84 | <0.001 |  |
| ^*^**Visceral adipose tissue, %** |  |  |  |  |  |  |  | 0.425 |
| Tertile 1 | Ref |  |  |  | Ref |  |  |  |
| Tertile 2 | 1.44 | 0.75, 2.78 | 0.267 |  | 3.36 | 0.78, 14.38 | 0.099 |  |
| Tertile 3 | 3.21 | 1.44, 7.15 | 0.006 |  | 6.47 | 2.02, 20.77 | 0.003 |  |

**Note:** BMI=body mass index; CI=confidence interval; IFG+IGT=impaired both fasting plasma glucose and glucose tolerance; FMI=fat mass index; LMI=lean mass index; OR=odds ratio; Ref=reference; WC=waist circumference.

All analyses were weighted and accounted for the complex survey design. Models were adjusted for sex, ethnicity, education, poverty income ratio, physical activity, smoking, alcohol drinking, and family history of diabetes. Models for fat distribution variables were additionally adjusted for height.

^*^ DEXA-derived body fat distribution variables were expressed as regional fat mass relative to total body fat mass, including appendicular adipose tissue (%), abdominal adipose tissue (%), gynoid adipose tissue (%) and visceral adipose tissue (%).

# Supplementary Tables 6 Stratified analyses by ethnicity

**Table S6.1** Logistic regression models for the associations of weight status, general adiposity, total lean mass, and body fat distribution with I-IFG by ethnicity

|  | **I-IFG** | | |  |  |  |  |  |  |  |  |  |
| --- | --- | --- | --- | --- | --- | --- | --- | --- | --- | --- | --- | --- |
|  | **Hispanic** | | |  | **Non-Hispanic White** | | |  | **Others** | | |  |
|  | **OR** | **95% CI** | **p value** |  | **OR** | **95% CI** | **p value** |  | **OR** | **95% CI** | **p value** | **p interaction** |
| **Weight Status** |  |  |  |  |  |  |  |  |  |  |  |  |
| **BMI kg/m^2^** |  |  |  |  |  |  |  |  |  |  |  | 0.770 |
| Normal | Ref |  |  |  | Ref |  |  |  | Ref |  |  |  |
| Underweight | 0.28 | 0.02, 4.23 | 0.348 |  | 0.45 | 0.06, 3.23 | 0.416 |  | 0.86 | 0.33, 2.23 | 0.742 |  |
| Overweight | 1.72 | 1.12, 2.66 | 0.016 |  | 1.48 | 0.98, 2.22 | 0.060 |  | 1.22 | 0.66, 2.28 | 0.512 |  |
| Obesity | 2.43 | 1.44, 4.09 | 0.002 |  | 2.77 | 1.78, 4.31 | <0.001 |  | 2.55 | 1.66, 3.91 | <0.001 |  |
| **General Adiposity** |  |  |  |  |  |  |  |  |  |  |  |  |
| **FMI, kg/m^2^** |  |  |  |  |  |  |  |  |  |  |  | 0.893 |
| Tertile 1 | Ref |  |  |  | Ref |  |  |  | Ref |  |  |  |
| Tertile 2 | 1.59 | 1.00, 2.54 | 0.051 |  | 1.51 | 1.05, 2.19 | 0.028 |  | 1.49 | 0.95, 2.35 | 0.083 |  |
| Tertile 3 | 3.29 | 1.89, 5.72 | <0.001 |  | 2.86 | 1.85, 4.43 | <0.001 |  | 2.23 | 1.46, 3.39 | <0.001 |  |
| **Total Lean Mass** |  |  |  |  |  |  |  |  |  |  |  |  |
| **LMI, kg/m^2^** |  |  |  |  |  |  |  |  |  |  |  | 0.158 |
| Tertile 1 | Ref |  |  |  | Ref |  |  |  | Ref |  |  |  |
| Tertile 2 | 1.59 | 0.99, 2.56 | 0.055 |  | 1.28 | 0.90, 1.81 | 0.169 |  | 1.25 | 0.83, 1.88 | 0.285 |  |
| Tertile 3 | 2.43 | 1.58, 3.73 | <0.001 |  | 2.73 | 1.85, 4.03 | <0.001 |  | 1.53 | 0.94, 2.49 | 0.088 |  |
| **Body Fat Distribution** |  |  |  |  |  |  |  |  |  |  |  |  |
| **WC, cm** |  |  |  |  |  |  |  |  |  |  |  | 0.892 |
| Normal | Ref |  |  |  | Ref |  |  |  | Ref |  |  |  |
| High-risk central obesity | 2.02 | 1.30, 3.14 | 0.003 |  | 1.86 | 1.20, 2.88 | 0.007 |  | 2.02 | 1.32, 3.11 | 0.002 |  |
| Very-high-risk central obesity | 3.50 | 2.05, 5.99 | <0.001 |  | 2.85 | 1.81, 4.48 | <0.001 |  | 2.76 | 1.80, 4.23 | <0.001 |  |
| ^*^**Appendicular adipose tissue, %** |  |  |  |  |  |  |  |  |  |  |  | 0.164 |
| Tertile 1 | Ref |  |  |  | Ref |  |  |  | Ref |  |  |  |
| Tertile 2 | 0.52 | 0.34, 0.79 | 0.003 |  | 0.51 | 0.33, 0.78 | 0.003 |  | 0.89 | 0.61, 1.29 | 0.526 |  |
| Tertile 3 | 0.32 | 0.21, 0.48 | <0.001 |  | 0.41 | 0.28, 0.60 | <0.001 |  | 0.50 | 0.34, 0.75 | 0.001 |  |
| ^*^**Gynoid adipose tissue, %** |  |  |  |  |  |  |  |  |  |  |  | 0.086 |
| Tertile 1 | Ref |  |  |  | Ref |  |  |  | Ref |  |  |  |
| Tertile 2 | 0.66 | 0.42, 1.06 | 0.083 |  | 0.52 | 0.35, 0.77 | 0.002 |  | 0.84 | 0.53, 1.32 | 0.437 |  |
| Tertile 3 | 0.33 | 0.22, 0.50 | <0.001 |  | 0.41 | 0.28, 0.59 | <0.001 |  | 0.73 | 0.49, 1.06 | 0.098 |  |
| ^*^**Abdominal adipose tissue, %** |  |  |  |  |  |  |  |  |  |  |  | 0.673 |
| Tertile 1 | Ref |  |  |  | Ref |  |  |  | Ref |  |  |  |
| Tertile 2 | 2.25 | 1.46, 3.46 | <0.001 |  | 1.46 | 0.98, 2.18 | 0.063 |  | 1.69 | 1.09, 2.62 | 0.022 |  |
| Tertile 3 | 2.41 | 1.41, 4.13 | 0.002 |  | 2.12 | 1.44, 3.13 | <0.001 |  | 2.19 | 1.41, 3.39 | <0.001 |  |
| ^*^**Visceral adipose tissue, %** |  |  |  |  |  |  |  |  |  |  |  | 0.469 |
| Tertile 1 | Ref |  |  |  | Ref |  |  |  | Ref |  |  |  |
| Tertile 2 | 1.41 | 0.78, 2.53 | 0.247 |  | 1.44 | 0.96, 2.17 | 0.074 |  | 1.15 | 0.78, 1.71 | 0.469 |  |
| Tertile 3 | 2.03 | 1.20, 3.43 | 0.010 |  | 1.64 | 1.11, 2.44 | 0.015 |  | 1.20 | 0.71, 2.02 | 0.483 |  |

**Note:** CI=confidence interval; FMI=fat mass index; LMI=lean mass index; I-IFG=isolated-impaired fasting glucose; OR=odds ratio; Ref=reference; WC=waist circumference.

All analyses were weighted and accounted for the complex survey design. Models were adjusted for age, sex, education, poverty income ratio, physical activity, smoking, alcohol drinking, and family history of diabetes. Models for fat distribution variables were additionally adjusted for height.

^*^ DEXA-derived body fat distribution variables were expressed as regional fat mass relative to total body fat mass, including appendicular adipose tissue (%), abdominal adipose tissue (%), gynoid adipose tissue (%) and visceral adipose tissue (%).

**Table S6.2** Logistic regression models for the associations of weight status, general adiposity, total lean mass, and body fat distribution with I-IGT by ethnicity

|  | **I-IGT** | | |  |  |  |  |  |  |  |  |  |
| --- | --- | --- | --- | --- | --- | --- | --- | --- | --- | --- | --- | --- |
|  | **Hispanic** | | |  | **Non-Hispanic White** | | |  | **Others** | | |  |
|  | **OR** | **95% CI** | **p value** |  | **OR** | **95% CI** | **p value** |  | **OR** | **95% CI** | **p value** | **p interaction** |
| **Weight Status** |  |  |  |  |  |  |  |  |  |  |  |  |
| **BMI kg/m^2^** |  |  |  |  |  |  |  |  |  |  |  | 0.262 |
| Normal | Ref |  |  |  | Ref |  |  |  | Ref |  |  |  |
| Underweight | ^†^0.00 | 0.00, 0.00 | <0.001 |  | 6.02 | 1.29, 28.06 | 0.024 |  | 2.02 | 0.43, 9.44 | 0.359 |  |
| Overweight | 0.91 | 0.36, 2.29 | 0.832 |  | 1.06 | 0.39, 2.86 | 0.909 |  | 1.15 | 0.49, 2.72 | 0.739 |  |
| Obesity | 3.06 | 1.14, 8.19 | 0.028 |  | 3.05 | 1.47, 6.33 | 0.004 |  | 1.58 | 0.68, 3.69 | 0.277 |  |
| **General Adiposity** |  |  |  |  |  |  |  |  |  |  |  |  |
| **FMI, kg/m^2^** |  |  |  |  |  |  |  |  |  |  |  | 0.175 |
| Tertile 1 | Ref |  |  |  | Ref |  |  |  | Ref |  |  |  |
| Tertile 2 | 2.60 | 0.67, 10.10 | 0.160 |  | 0.63 | 0.27, 1.49 | 0.283 |  | 1.23 | 0.69, 2.17 | 0.474 |  |
| Tertile 3 | 4.46 | 1.05, 18.92 | 0.043 |  | 1.89 | 1.02, 3.50 | 0.043 |  | 1.40 | 0.65, 3.03 | 0.376 |  |
| **Total Lean Mass** |  |  |  |  |  |  |  |  |  |  |  |  |
| **LMI, kg/m^2^** |  |  |  |  |  |  |  |  |  |  |  | 0.180 |
| Tertile 1 | Ref |  |  |  | Ref |  |  |  | Ref |  |  |  |
| Tertile 2 | 1.08 | 0.33, 3.45 | 0.899 |  | 1.21 | 0.48, 3.06 | 0.682 |  | 0.62 | 0.29, 1.31 | 0.200 |  |
| Tertile 3 | 3.05 | 1.02, 9.14 | 0.047 |  | 2.03 | 0.95, 4.32 | 0.067 |  | 0.60 | 0.29, 1.23 | 0.156 |  |
| **Body Fat Distribution** |  |  |  |  |  |  |  |  |  |  |  |  |
| **WC, cm** |  |  |  |  |  |  |  |  |  |  |  | 0.245 |
| Normal | Ref |  |  |  | Ref |  |  |  | Ref |  |  |  |
| High-risk central obesity | 1.59 | 0.33, 7.66 | 0.546 |  | 0.88 | 0.30, 2.64 | 0.817 |  | 1.63 | 0.64, 4.19 | 0.294 |  |
| Very-high-risk central obesity | 5.26 | 1.17, 23.71 | 0.032 |  | 1.51 | 0.76, 3.03 | 0.230 |  | 1.41 | 0.54, 3.69 | 0.470 |  |
| ^*^**Appendicular adipose tissue, %** |  |  |  |  |  |  |  |  |  |  |  | 0.093 |
| Tertile 1 | Ref |  |  |  | Ref |  |  |  | Ref |  |  |  |
| Tertile 2 | 0.57 | 0.27, 1.21 | 0.135 |  | 1.28 | 0.57, 2.90 | 0.538 |  | 0.74 | 0.33, 1.65 | 0.448 |  |
| Tertile 3 | 0.17 | 0.03, 0.82 | 0.029 |  | 1.15 | 0.55, 2.39 | 0.706 |  | 0.47 | 0.19, 1.15 | 0.096 |  |
| ^*^**Gynoid adipose tissue, %** |  |  |  |  |  |  |  |  |  |  |  | 0.271 |
| Tertile 1 | Ref |  |  |  | Ref |  |  |  | Ref |  |  |  |
| Tertile 2 | 0.32 | 0.13, 0.79 | 0.015 |  | 1.01 | 0.50, 2.04 | 0.985 |  | 0.66 | 0.29, 1.52 | 0.315 |  |
| Tertile 3 | 0.38 | 0.15, 0.98 | 0.045 |  | 0.52 | 0.23, 1.17 | 0.109 |  | 0.33 | 0.15, 0.71 | 0.006 |  |
| ^*^**Abdominal adipose tissue, %** |  |  |  |  |  |  |  |  |  |  |  | 0.286 |
| Tertile 1 | Ref |  |  |  | Ref |  |  |  | Ref |  |  |  |
| Tertile 2 | 5.57 | 0.98, 31.76 | 0.053 |  | 1.00 | 0.42, 2.35 | 0.995 |  | 1.89 | 0.79, 4.56 | 0.148 |  |
| Tertile 3 | 3.87 | 0.72, 20.78 | 0.110 |  | 1.01 | 0.45, 2.28 | 0.974 |  | 2.32 | 0.88, 6.15 | 0.087 |  |
| ^*^**Visceral adipose tissue, %** |  |  |  |  |  |  |  |  |  |  |  | 0.433 |
| Tertile 1 | Ref |  |  |  | Ref |  |  |  | Ref |  |  |  |
| Tertile 2 | 2.22 | 0.73, 6.75 | 0.153 |  | 1.28 | 0.48, 3.39 | 0.612 |  | 1.29 | 0.57, 2.92 | 0.525 |  |
| Tertile 3 | 2.64 | 0.91, 7.66 | 0.073 |  | 1.05 | 0.43, 2.56 | 0.906 |  | 2.08 | 0.66, 6.57 | 0.205 |  |

**Note:** CI=confidence interval; FMI=fat mass index; I-IGT=isolated-impaired glucose tolerance; OR=odds ratio; Ref=reference; WC=waist circumference.

All analyses were weighted and accounted for the complex survey design. Models were adjusted for age, sex, education, poverty income ratio, physical activity, smoking, alcohol drinking, and family history of diabetes. Models for fat distribution variables were additionally adjusted for height.

^*^ DEXA-derived body fat distribution variables were expressed as regional fat mass relative to total body fat mass, including appendicular adipose tissue (%), abdominal adipose tissue (%), gynoid adipose tissue (%) and visceral adipose tissue (%).

^†^ Estimates of 0.00 with 95% CI 0.00–0.00 indicate sparse data

**Table S6.3** Logistic regression models for the associations of weight status, general adiposity, total lean mass, and body fat distribution with IFG+IGT by ethnicity

|  | **IFG+IGT** | | |  |  |  |  |  |  |  |  |  |
| --- | --- | --- | --- | --- | --- | --- | --- | --- | --- | --- | --- | --- |
|  | **Hispanic** | | |  | **Non-Hispanic White** | | |  | **Others** | | |  |
|  | **OR** | **95% CI** | **p value** |  | **OR** | **95% CI** | **p value** |  | **OR** | **95% CI** | **p value** | **p interaction** |
| **Weight Status** |  |  |  |  |  |  |  |  |  |  |  |  |
| **BMI kg/m^2^** |  |  |  |  |  |  |  |  |  |  |  | 0.340 |
| Normal | Ref |  |  |  | Ref |  |  |  | Ref |  |  |  |
| Underweight | 1.69 | 0.12, 24.24 | 0.690 |  | ^†^0.00 | 0.00, 0.00 | <0.001 |  | 1.54 | 0.37, 6.49 | 0.542 |  |
| Overweight | 6.22 | 1.71, 22.62 | 0.007 |  | 2.87 | 1.26, 6.54 | 0.014 |  | 1.41 | 0.58, 3.42 | 0.436 |  |
| Obesity | 11.83 | 3.37, 41.60 | <0.001 |  | 8.35 | 3.61, 19.35 | <0.001 |  | 4.38 | 2.09, 9.19 | <0.001 |  |
| **General Adiposity** |  |  |  |  |  |  |  |  |  |  |  |  |
| **FMI, kg/m^2^** |  |  |  |  |  |  |  |  |  |  |  | 0.431 |
| Tertile 1 | Ref |  |  |  | Ref |  |  |  | Ref |  |  |  |
| Tertile 2 | 6.31 | 1.77, 22.57 | 0.006 |  | 5.49 | 1.72, 17.57 | 0.006 |  | 1.84 | 0.87, 3.90 | 0.106 |  |
| Tertile 3 | 17.19 | 4.77, 61.98 | <0.001 |  | 12.59 | 3.70, 42.89 | <0.001 |  | 4.41 | 2.37, 8.22 | <0.001 |  |
| **Total Lean Mass** |  |  |  |  |  |  |  |  |  |  |  |  |
| **LMI, kg/m^2^** |  |  |  |  |  |  |  |  |  |  |  | 0.080 |
| Tertile 1 | Ref |  |  |  | Ref |  |  |  | Ref |  |  |  |
| Tertile 2 | 2.43 | 0.81, 7.29 | 0.108 |  | 1.95 | 0.79, 4.83 | 0.144 |  | 1.33 | 0.61, 2.91 | 0.456 |  |
| Tertile 3 | 7.39 | 3.02, 18.07 | <0.001 |  | 9.84 | 5.04, 19.23 | <0.001 |  | 2.03 | 1.00, 4.13 | 0.051 |  |
| **Body Fat Distribution** |  |  |  |  |  |  |  |  |  |  |  |  |
| **WC, cm** |  |  |  |  |  |  |  |  |  |  |  | 0.063 |
| Normal | Ref |  |  |  | Ref |  |  |  | Ref |  |  |  |
| High-risk central obesity | 4.91 | 1.94, 12.40 | 0.002 |  | 7.93 | 1.83, 34.27 | 0.007 |  | 2.73 | 1.27, 5.85 | 0.012 |  |
| Very-high-risk central obesity | 14.99 | 4.92, 45.65 | <0.001 |  | 28.05 | 7.72, 101.92 | <0.001 |  | 4.01 | 2.00, 8.04 | <0.001 |  |
| ^*^**Appendicular adipose tissue, %** |  |  |  |  |  |  |  |  |  |  |  | 0.133 |
| Tertile 1 | Ref |  |  |  | Ref |  |  |  | Ref |  |  |  |
| Tertile 2 | 0.43 | 0.20, 0.92 | 0.030 |  | 0.22 | 0.12, 0.41 | <0.001 |  | 0.60 | 0.32, 1.16 | 0.125 |  |
| Tertile 3 | 0.30 | 0.12, 0.74 | 0.011 |  | 0.22 | 0.10, 0.48 | <0.001 |  | 0.28 | 0.14, 0.58 | 0.001 |  |
| ^*^**Gynoid adipose tissue, %** |  |  |  |  |  |  |  |  |  |  |  | 0.155 |
| Tertile 1 | Ref |  |  |  | Ref |  |  |  | Ref |  |  |  |
| Tertile 2 | 0.52 | 0.27, 0.99 | 0.045 |  | 0.23 | 0.10, 0.52 | 0.001 |  | 0.29 | 0.15, 0.57 | <0.001 |  |
| Tertile 3 | 0.14 | 0.05, 0.37 | <0.001 |  | 0.21 | 0.08, 0.53 | 0.002 |  | 0.39 | 0.22, 0.72 | 0.004 |  |
| ^*^**Abdominal adipose tissue, %** |  |  |  |  |  |  |  |  |  |  |  | 0.512 |
| Tertile 1 | Ref |  |  |  | Ref |  |  |  | Ref |  |  |  |
| Tertile 2 | 2.44 | 0.96, 6.22 | 0.060 |  | 2.33 | 0.90, 6.04 | 0.081 |  | 2.08 | 1.06, 4.10 | 0.035 |  |
| Tertile 3 | 4.14 | 1.25, 13.72 | 0.022 |  | 5.47 | 2.09, 14.36 | 0.001 |  | 2.76 | 1.44, 5.28 | 0.003 |  |
| ^*^**Visceral adipose tissue, %** |  |  |  |  |  |  |  |  |  |  |  | 0.498 |
| Tertile 1 | Ref |  |  |  | Ref |  |  |  | Ref |  |  |  |
| Tertile 2 | 1.57 | 0.53, 4.65 | 0.400 |  | 2.17 | 0.79, 5.97 | 0.129 |  | 0.95 | 0.42, 2.14 | 0.902 |  |
| Tertile 3 | 4.26 | 1.11, 16.31 | 0.035 |  | 4.31 | 1.66, 11.20 | 0.004 |  | 2.37 | 0.93, 6.06 | 0.070 |  |

**Note:** CI=confidence interval; IFG+IGT=impaired both fasting plasma glucose and glucose tolerance; FMI=fat mass index; LMI=lean mass index; OR=odds ratio; Ref=reference; WC=waist circumference.

All analyses were weighted and accounted for the complex survey design. Models were adjusted for age, sex, education, poverty income ratio, physical activity, smoking, alcohol drinking, and family history of diabetes. Models for fat distribution variables were additionally adjusted for height.

^*^ DEXA-derived body fat distribution variables were expressed as regional fat mass relative to total body fat mass, including appendicular adipose tissue (%), abdominal adipose tissue (%), gynoid adipose tissue (%) and visceral adipose tissue (%).

^†^ Estimates of 0.00 with 95% CI 0.00–0.00 indicate sparse data

# Supplementary Tables 7 Stratified analyses by weight status

**Table S7.1** Logistic regression models for the associations of body fat distribution with I-IFG by weight status

|  | **I-IFG** |  |  |  |  |  |  |  |
| --- | --- | --- | --- | --- | --- | --- | --- | --- |
|  | **Non-overweight** | | |  | **Overweight or Obesity** | | |  |
|  | **OR** | **95% CI** | **p value** |  | **OR** | **95% CI** | **p value** | **p interaction** |
| **Body Fat Distribution** |  |  |  |  |  |  |  |  |
| **WC, cm** |  |  |  |  |  |  |  | 0.699 |
| Normal | Ref |  |  |  | Ref |  |  |  |
| High-risk central obesity | 2.17 | 1.07, 4.39 | 0.032 |  | 1.82 | 1.25, 2.64 | 0.003 |  |
| Very-high-risk central obesity | 2.66 | 0.82, 8.59 | 0.099 |  | 2.51 | 1.61, 3.91 | <0.001 |  |
| ^*^**Appendicular adipose tissue, %** |  |  |  |  |  |  |  | 0.953 |
| Tertile 1 | Ref |  |  |  | Ref |  |  |  |
| Tertile 2 | 0.61 | 0.30, 1.21 | 0.152 |  | 0.58 | 0.42, 0.80 | 0.002 |  |
| Tertile 3 | 0.49 | 0.29, 0.84 | 0.011 |  | 0.51 | 0.34, 0.76 | 0.002 |  |
| ^*^**Gynoid adipose tissue, %** |  |  |  |  |  |  |  | 0.668 |
| Tertile 1 | Ref |  |  |  | Ref |  |  |  |
| Tertile 2 | 0.58 | 0.27, 1.25 | 0.157 |  | 0.66 | 0.50, 0.87 | 0.005 |  |
| Tertile 3 | 0.59 | 0.28, 1.23 | 0.150 |  | 0.52 | 0.37, 0.74 | <0.001 |  |
| ^*^**Abdominal adipose tissue, %** |  |  |  |  |  |  |  | 0.269 |
| Tertile 1 | Ref |  |  |  | Ref |  |  |  |
| Tertile 2 | 0.99 | 0.60, 1.63 | 0.953 |  | 1.51 | 0.96, 2.38 | 0.074 |  |
| Tertile 3 | 1.28 | 0.72, 2.29 | 0.385 |  | 1.88 | 1.22, 2.91 | 0.006 |  |
| ^*^**Visceral adipose tissue, %** |  |  |  |  |  |  |  | 0.419 |
| Tertile 1 | Ref |  |  |  | Ref |  |  |  |
| Tertile 2 | 1.09 | 0.67, 1.77 | 0.720 |  | 1.44 | 1.07, 1.95 | 0.018 |  |
| Tertile 3 | 1.50 | 0.75, 3.02 | 0.245 |  | 1.38 | 1.00, 1.90 | 0.051 |  |

**Note:** CI=confidence interval; I-IFG=isolated-impaired fasting glucose; OR=odds ratio; Ref=reference; WC=waist circumference.

All analyses were weighted and accounted for the complex survey design. Models were adjusted for age, sex, ethnicity, education, poverty income ratio, physical activity, smoking, alcohol drinking, family history of diabetes, and height.

^*^ DEXA-derived body fat distribution variables were expressed as regional fat mass relative to total body fat mass, including appendicular adipose tissue (%), abdominal adipose tissue (%), gynoid adipose tissue (%) and visceral adipose tissue (%).

**Table S7.2** Logistic regression models for the associations of body fat distribution with I-IGT by weight status

|  | **I-IGT** |  |  |  |  |  |  |  |
| --- | --- | --- | --- | --- | --- | --- | --- | --- |
|  | **Non-overweight** | | |  | **Overweight or Obesity** | | |  |
|  | **OR** | **95% CI** | **p value** |  | **OR** | **95% CI** | **p value** | **p interaction** |
| **Body Fat Distribution** |  |  |  |  |  |  |  |  |
| **WC, cm** |  |  |  |  |  |  |  | 0.063 |
| Normal | Ref |  |  |  | Ref |  |  |  |
| High-risk central obesity | 1.06 | 0.35, 3.25 | 0.914 |  | 1.53 | 0.52, 4.46 | 0.428 |  |
| Very-high-risk central obesity | 0.26 | 0.05, 1.50 | 0.128 |  | 3.39 | 1.36, 8.47 | 0.010 |  |
| ^*^**Appendicular adipose tissue, %** |  |  |  |  |  |  |  | 0.181 |
| Tertile 1 | Ref |  |  |  | Ref |  |  |  |
| Tertile 2 | 1.74 | 0.66, 4.58 | 0.250 |  | 0.99 | 0.54, 1.83 | 0.972 |  |
| Tertile 3 | 1.35 | 0.47, 3.85 | 0.561 |  | 0.75 | 0.35, 1.61 | 0.445 |  |
| ^*^**Gynoid adipose tissue, %** |  |  |  |  |  |  |  | 0.142 |
| Tertile 1 | Ref |  |  |  | Ref |  |  |  |
| Tertile 2 | 1.41 | 0.51, 3.92 | 0.496 |  | 0.70 | 0.41, 1.20 | 0.189 |  |
| Tertile 3 | 0.80 | 0.26, 2.43 | 0.682 |  | 0.35 | 0.17, 0.72 | 0.006 |  |
| ^*^**Abdominal adipose tissue, %** |  |  |  |  |  |  |  | 0.107 |
| Tertile 1 | Ref |  |  |  | Ref |  |  |  |
| Tertile 2 | 0.99 | 0.44, 2.21 | 0.976 |  | 1.80 | 0.64, 5.06 | 0.257 |  |
| Tertile 3 | 0.60 | 0.24, 1.50 | 0.264 |  | 1.56 | 0.57, 4.26 | 0.377 |  |
| ^*^**Visceral adipose tissue, %** |  |  |  |  |  |  |  | 0.064 |
| Tertile 1 | Ref |  |  |  | Ref |  |  |  |
| Tertile 2 | 1.50 | 0.62, 3.59 | 0.354 |  | 1.50 | 0.65, 3.43 | 0.327 |  |
| Tertile 3 | 0.57 | 0.22, 1.49 | 0.238 |  | 1.48 | 0.67, 3.27 | 0.323 |  |

**Note:** CI=confidence interval; I-IGT=isolated-impaired glucose tolerance; OR=odds ratio; Ref=reference; WC=waist circumference.

All analyses were weighted and accounted for complex survey design. Models were adjusted for age, sex, ethnicity, education, poverty income ratio, physical activity, smoking, alcohol drinking, family history of diabetes, and height.

^*^ DEXA-derived body fat distribution variables were expressed as regional fat mass relative to total body fat mass, including appendicular adipose tissue (%), abdominal adipose tissue (%), gynoid adipose tissue (%) and visceral adipose tissue (%).

**Table S7.3** Logistic regression models for the associations of weight status, general adiposity, total lean mass, and body fat distribution with IFG+IGT by weight status

|  | **IFG+IGT** |  |  |  |  |  |  |  |
| --- | --- | --- | --- | --- | --- | --- | --- | --- |
|  | **Non-overweight** | | |  | **Overweight or Obesity** | | |  |
|  | **OR** | **95% CI** | **p value** |  | **OR** | **95% CI** | **p value** | **p interaction** |
| **Body Fat Distribution** |  |  |  |  |  |  |  |  |
| **WC, cm** |  |  |  |  |  |  |  | 0.706 |
| Normal | Ref |  |  |  | Ref |  |  |  |
| High-risk central obesity | 6.08 | 1.66, 22.32 | 0.008 |  | 3.85 | 1.75, 8.50 | 0.002 |  |
| Very-high-risk central obesity | 14.59 | 2.61, 81.52 | 0.003 |  | 10.09 | 4.85, 21.02 | <0.001 |  |
| ^*^**Appendicular adipose tissue, %** |  |  |  |  |  |  |  | 0.381 |
| Tertile 1 | Ref |  |  |  | Ref |  |  |  |
| Tertile 2 | 0.51 | 0.19, 1.37 | 0.176 |  | 0.33 | 0.22, 0.51 | <0.001 |  |
| Tertile 3 | 0.28 | 0.11, 0.69 | 0.008 |  | 0.39 | 0.21, 0.69 | 0.002 |  |
| ^*^**Gynoid adipose tissue, %** |  |  |  |  |  |  |  | 0.108 |
| Tertile 1 | Ref |  |  |  | Ref |  |  |  |
| Tertile 2 | 0.15 | 0.05, 0.44 | 0.001 |  | 0.40 | 0.24, 0.67 | 0.001 |  |
| Tertile 3 | 0.55 | 0.21, 1.45 | 0.219 |  | 0.30 | 0.15, 0.62 | 0.002 |  |
| ^*^**Abdominal adipose tissue, %** |  |  |  |  |  |  |  | 0.602 |
| Tertile 1 | Ref |  |  |  | Ref |  |  |  |
| Tertile 2 | 1.28 | 0.38, 4.24 | 0.681 |  | 1.50 | 0.77, 2.93 | 0.226 |  |
| Tertile 3 | 4.53 | 1.82, 11.29 | 0.002 |  | 2.43 | 1.17, 5.06 | 0.019 |  |
| ^*^**Visceral adipose tissue, %** |  |  |  |  |  |  |  | 0.639 |
| Tertile 1 | Ref |  |  |  | Ref |  |  |  |
| Tertile 2 | 2.16 | 0.57, 8.16 | 0.246 |  | 1.41 | 0.69, 2.90 | 0.333 |  |
| Tertile 3 | 3.42 | 1.07, 10.99 | 0.040 |  | 2.49 | 1.24, 4.99 | 0.012 |  |

**Note:** CI=confidence interval; IFG+IGT=impaired both fasting plasma glucose and glucose tolerance; OR=odds ratio; Ref=reference; WC=waist circumference.

All analyses were weighted and accounted for complex survey design. Models were adjusted for age, sex, ethnicity, education, poverty income ratio, physical activity, smoking, alcohol drinking, family history of diabetes, and height.

^*^ DEXA-derived body fat distribution variables were expressed as regional fat mass relative to total body fat mass, including appendicular adipose tissue (%), abdominal adipose tissue (%), gynoid adipose tissue (%) and visceral adipose tissue (%).

# Supplementary Figure 1 Flowchart of the study population selection process

**

**

**Figure S1** Flowchart of the study population selection process

# Supplementary Figure 2 Restricted cubic spline analyses

**
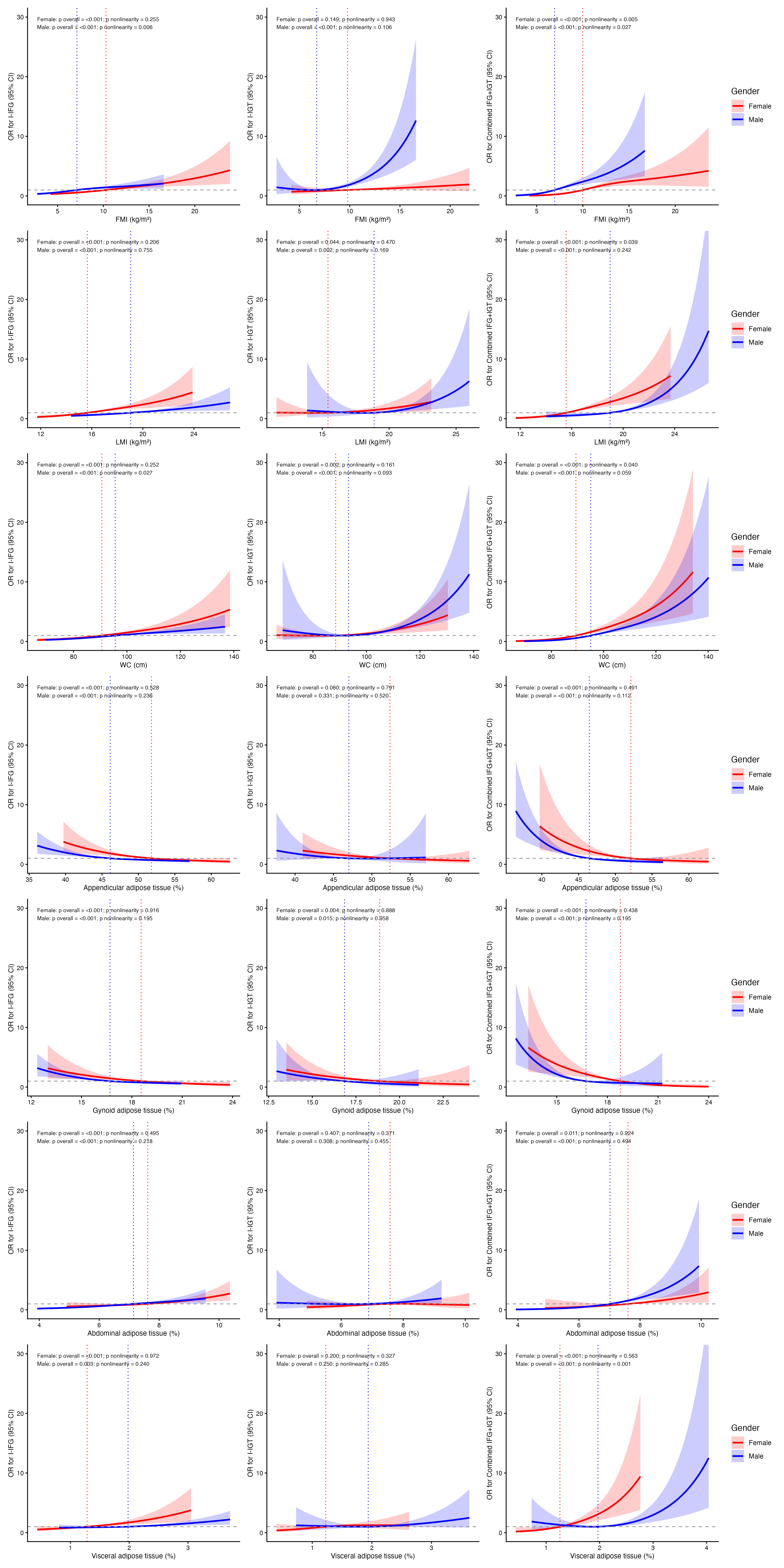
**

**Figure S2** Restricted cubic spline analyses of general adiposity, total lean mass, body fat distribution with three prediabetes phenotypes by sex

**Note:** BMI=body mass index; FMI=fat mass index; LMI=lean mass index; WC=waist circumference. The solid curved lines represent odds ratios for three pre-diabetes phenotypes, and the shading areas represent 95% confidence intervals. All analyses were weighted and accounted for complex survey design. Models were adjusted for age, ethnicity, education, poverty income ratio, physical activity, smoking, alcohol drinking, and family history of diabetes. Models for body fat distribution variables were additionally adjusted for height. DEXA-derived body fat distribution variables were expressed as regional fat mass relative to total body fat mass, including appendicular adipose tissue (%), abdominal adipose tissue (%), gynoid adipose tissue (%) and visceral adipose tissue (%).
